# A global multiproxy database for temperature reconstructions of the Common Era

**DOI:** 10.1038/sdata.2017.88

**Published:** 2017-07-11

**Authors:** PAGES2k Consortium, Julien Emile-Geay, Nicholas P. McKay, Darrell S. Kaufman, Lucien von Gunten, Jianghao Wang, Kevin J. Anchukaitis, Nerilie J. Abram, Jason A. Addison, Mark A.J. Curran, Michael N. Evans, Benjamin J. Henley, Zhixin Hao, Belen Martrat, Helen V. McGregor, Raphael Neukom, Gregory T. Pederson, Barbara Stenni, Kaustubh Thirumalai, Johannes P. Werner, Chenxi Xu, Dmitry V. Divine, Bronwyn C. Dixon, Joelle Gergis, Ignacio A. Mundo, Takeshi Nakatsuka, Steven J. Phipps, Cody C. Routson, Eric J. Steig, Jessica E. Tierney, Jonathan J. Tyler, Kathryn J. Allen, Nancy A.N. Bertler, Jesper Björklund, Brian M. Chase, Min-Te Chen, Ed Cook, Rixt de Jong, Kristine L. DeLong, Daniel A. Dixon, Alexey A. Ekaykin, Vasile Ersek, Helena L. Filipsson, Pierre Francus, Mandy B. Freund, Massimo Frezzotti, Narayan P. Gaire, Konrad Gajewski, Quansheng Ge, Hugues Goosse, Anastasia Gornostaeva, Martin Grosjean, Kazuho Horiuchi, Anne Hormes, Katrine Husum, Elisabeth Isaksson, Selvaraj Kandasamy, Kenji Kawamura, K. Halimeda Kilbourne, Nalan Koç, Guillaume Leduc, Hans W. Linderholm, Andrew M. Lorrey, Vladimir Mikhalenko, P. Graham Mortyn, Hideaki Motoyama, Andrew D. Moy, Robert Mulvaney, Philipp M. Munz, David J. Nash, Hans Oerter, Thomas Opel, Anais J. Orsi, Dmitriy V. Ovchinnikov, Trevor J. Porter, Heidi A. Roop, Casey Saenger, Masaki Sano, David Sauchyn, Krystyna M. Saunders, Marit-Solveig Seidenkrantz, Mirko Severi, Xuemei Shao, Marie-Alexandrine Sicre, Michael Sigl, Kate Sinclair, Scott St. George, Jeannine-Marie St. Jacques, Meloth Thamban, Udya Kuwar Thapa, Elizabeth R. Thomas, Chris Turney, Ryu Uemura, Andre E. Viau, Diana O. Vladimirova, Eugene R. Wahl, James W.C. White, Zicheng Yu, Jens Zinke

**Affiliations:** 1 Department of Earth Sciences and Center for Applied Mathematical Sciences, University of Southern California, Los Angeles, CA 90089, USA; 2 School of Earth Sciences and Environmental Sustainability, Northern Arizona University, Flagstaff, AZ 86001, USA; 3 PAGES International Project Office, Bern 3012, Switzerland; 4 The Mathworks Inc., Natick, MA 01760, USA; 5 School of Geography and Development and Laboratory of Tree Ring Research, University of Arizona, Tucson, AZ 85721, USA; 6 Research School of Earth Sciences and the ARC Centre of Excellence for Climate System Science, The Australian National University, Canberra, ACT 2601, Australia; 7 U.S. Geological Survey, Menlo Park, CA 94025, USA; 8 Australian Antarctic Division, Kingston, TAS 7050, Australia; 9 Antarctic Climate and Ecosystems CRC, University of Tasmania, Hobart, TAS 7050, Australia; 10 Department of Geology and Earth System Science Interdisciplinary Center, University of Maryland, College Park, MD 20742, USA; 11 School of Earth Sciences, University of Melbourne, Melbourne, VIC 3010, Australia; 12 The Institute of Geographic Sciences and Natural Resources Research, Chinese Academy of Sciences, Beijing 100101, China; 13 Department of Environmental Chemistry, Institute of Environmental Assessment and Water Research, Spanish Council for Scientific Research, Barcelona 08034, Spain; 14 Department of Earth Sciences, University of Cambridge, Cambridge CB2 3EQ, UK; 15 School of Earth and Environmental Sciences, University of Wollongong, Wollongong, NSW 2522, Australia; 16 Oeschger Centre for Climate Change Research and Institute of Geography, University of Bern, Bern 3012, Switzerland; 17 U.S. Geological Survey, Northern Rocky Mountain Science Center, Bozeman, MT 59715, USA; 18 Department of Environmental Sciences, Informatics and Statistics, Ca’ Foscari University of Venice, Venice 30170, Italy; 19 Institute for Geophysics, Jackson School of Geosciences, University of Texas at Austin, Austin, Texas 78758, USA; 20 Department for Earth Science and Bjerknes Centre for Climate Research, University of Bergen, Bergen 5020, Norway; 21 Institute of Geology and Geophysics, Chinese Academy of Sciences, Beijing 100029, China; 22 Norwegian Polar Institute, Fram Centre, Tromsø 9296, Norway; 23 Department of Mathematics and Statistics, Faculty of Science and Technology, University of Tromsø—The Arctic University of Norway, Tromsø 9037, Norway; 24 School of Geography, The University of Melbourne, Melbourne, VIC 3010, Australia; 25 School of Earth Sciences and Australian Research Council Centre of Excellence for Climate System Science, University of Melbourne, Melbourne, VIC 3010, Australia; 26 IANIGLA-CONICET and Facultad de Ciencias Exactas y Naturales, Universidad Nacional de Cuyo, Mendoza M5502IRA, Argentina; 27 Research Institute for Humanity and Nature, Kyoto 603-8047, Japan; 28 Institute for Marine and Antarctic Studies, University of Tasmania, Hobart, TAS 7001, Australia; 29 Quaternary Research Center and Department of Earth and Space Sciences, University of Washington, Seattle, WA 98195, USA; 30 Department of Geosciences, University of Arizona, Tucson, AZ 85721, USA; 31 Department of Earth Sciences and Sprigg Geobiology Centre, The University of Adelaide, Adelaide, SA 5005, Australia; 32 School of Ecosystem and Forest Sciences, University of Melbourne, Melbourne, VIC 3121, Australia; 33 Joint Antarctic Research Institute, Victoria University of Wellington and GNS Science, Wellington 6012, New Zealand; 34 Swiss Federal Research Institute WSL, Birmensdorf 8903, Switzerland; 35 Centre National de Recherche Scientifique, UMR 5554, Institut des Sciences de l’Evolution-Montpellier, Université Montpellier, Montpellier, cedex 5 34095, France; 36 Institute of Applied Geosciences, National Taiwan Ocean University, Keelung 20224, Taiwan; 37 Lamont-Doherty Earth Observatory, Palisades, NY 10964, USA; 38 Department of Geography and Anthropology, Louisiana State University, Baton Rouge, LA 70803, USA; 39 Climate Change Institute, University of Maine, Orono, ME 04469, USA; 40 Arctic and Antarctic Research Institute, St Petersburg 199397, Russia; 41 Institute of Earth Sciences, St Petersburg State University, St Petersburg 199178, Russia; 42 Department of Geography, Northumbria University, Newcastle upon Tyne NE1 8ST, UK; 43 Department of Geology, Lund University, Lund SE-223 62, Sweden; 44 Institut National de la Recherche Scientifique, Centre Eau Terre Environment, Québec, QC G1K 9A9, Canada; 45 ENEA, CR Casaccia, Rome 00123, Italy; 46 Faculty of Science, Nepal Academy of Science and Technology, Lalitpur, Nepal; 47 Central Department of Environmental Science, Tribhuvan University, Kathmandu, Nepal; 48 Department of Geography, Environment and Geomatics, University of Ottawa, Ottawa, ON K1N 6N5, Canada; 49 Earth and Life Institute, Université catholique de Louvain, Louvain-la-Neuve 1348, Belgium; 50 Institute of Geophysics of the Urals Branch of RAS, Yekaterinburg, Russian Federation; 51 Graduate School of Science and Technology, Hirosaki University, Aomori 036-8561, Japan; 52 Department of Earth Sciences, The Faculty of Science, University of Gothenburg, Göteborg SE-405 30, Sweden; 53 State Key Laboratory of Marine Environmental Science and Department of Geological Oceanography, Xiamen University, Xiamen 361102, China; 54 National Institute of Polar Research and Department of Polar Science, Tachikawa, Tokyo 190-8518, Japan; 55 Institute of Biogeosciences, Japan Agency for Marine-Earth Science and Technology, Yokosuka 237- 0061, Japan; 56 University of Maryland Center for Environmental Science, Chesapeake Biological Laboratory, Solomons, Maryland 20688, USA; 57 Aix Marseille Université, CNRS, IRD, CEREGE UM34, Aix-en-Provence, Cedex 4 13545, France; 58 Department of Earth Sciences, University of Gothenburg, Göteborg SE-405 30, Sweden; 59 National Institute of Water and Atmospheric Research, Auckland Central 1010, New Zealand; 60 Institute of Geography, Russian Academy of Sciences, Moscow 119017, Russia; 61 Institute of Environmental Science and Technology, and Department of Geography, Universitat Autonoma de Barcelona, Bellaterra 08193, Spain; 62 National Institute of Polar Research, Research Organization of Information and Systems, Midoricho 10-3, Tachikawa, Tokyo, Japan; 63 British Antarctic Survey, Cambridge CB3 0ET, UK; 64 Eberhard Karls Universität Tübingen, Tübingen 72074, Germany; 65 School of Environment and Technology, University of Brighton, Brighton BN2 4GJ, UK; 66 School of Geography, Archaeology and Environmental Studies, University of the Witwatersrand, Wits 2050, South Africa; 67 Alfred Wegener Institute Helmholtz Centre for Polar and Marine Research, Bremerhaven 27515, Germany; 68 Alfred Wegener Institute Helmholtz Centre for Polar and Marine Research, Potsdam 14473, Germany; 69 Laboratoire des Sciences du Climat et de l’Environnement, Gif-sur-Yvette 91191, France; 70 Sukachev Institute of Forest of the Siberian Branch of the Russian Academy of the Sciences, Krasnoyarsk 660036, Russia; 71 Department of Geography, University of Toronto, Mississauga, ON L5L 1C6, Canada; 72 Department of Geology, University of Buffalo, NY 14260, USA; 73 Joint Institute for the Study of the Atmosphere and Ocean, University of Washington, Seattle, WA 98105, USA; 74 Australian Nuclear Science and Technology Organisation, Lucas Heights, NSW 2234, Australia; 75 Oeschger Centre for Climate Change Research and Institute of Geography, University of Bern, Bern 3012, Switzerland; 76 Centre for Past Climate Studies, and Arctic Research Centre, Department of Geoscience, Aarhus University, Aarhus C DK-8000, Denmark; 77 Chemistry Department, University of Florence, Sesto Fiorentino, Italy; 78 LOCEAN, Sorbonne Universités, Case 100, Paris F-75005, France; 79 Laboratory of Environmental Chemistry, Paul Scherrer Institut, Villigen, PSI Ost 5232, Switzerland; 80 Applied Aquatic Research Ltd., Calgary, AB T3C 0K3, Canada; 81 Department of Geography, Environment and Society, University of Minnesota, Minneapolis, MN 55455, USA; 82 Prairie Adaptation Research Collaborative, University of Regina, Regina, SK S4S 0A2, Canada; 83 Geography, Planning and Environment, Concordia University, Montreal, QC H3G 1M8, Canada; 84 National Centre for Antarctic and Ocean Research, Goa 403804, India; 85 Climate Change Research Centre, School of Biological, Earth and Environmental Sciences, University of New South Wales, Sydney, NSW 2052, Australia; 86 Department of Chemistry, Biology, and Marine Science, Faculty of Science, University of the Ryukyus, Nishihara, Okinawa 903-0213, Japan; 87 NOAA’s National Centers for Environmental Information, World Data Service for Paleoclimatology, Boulder, CO 80305, USA; 88 Institute of Arctic and Alpine Research, University of Colorado, Boulder, Colorado 80309, USA; 89 Department of Earth and Environmental Sciences, Lehigh University, Bethlehem, PA 18015, USA; 90 Department of Environment and Agriculture, Bentley, WA 6845, Australia; 91 Australian Institute of Marine Science, Townsville, QLD 4810, Australia; 92 Institute of Geological Sciences, Paleontology, Freie Universität Berlin, Berlin 12249, Germany

**Keywords:** Palaeoclimate, Climate change

## Abstract

Reproducible climate reconstructions of the Common Era (1 CE to present) are key to placing industrial-era warming into the context of natural climatic variability. Here we present a community-sourced database of temperature-sensitive proxy records from the PAGES2k initiative. The database gathers 692 records from 648 locations, including all continental regions and major ocean basins. The records are from trees, ice, sediment, corals, speleothems, documentary evidence, and other archives. They range in length from 50 to 2000 years, with a median of 547 years, while temporal resolution ranges from biweekly to centennial. Nearly half of the proxy time series are significantly correlated with HadCRUT4.2 surface temperature over the period 1850–2014. Global temperature composites show a remarkable degree of coherence between high- and low-resolution archives, with broadly similar patterns across archive types, terrestrial versus marine locations, and screening criteria. The database is suited to investigations of global and regional temperature variability over the Common Era, and is shared in the Linked Paleo Data (LiPD) format, including serializations in Matlab, R and Python.

## Background & Summary

Since the pioneering work of D’Arrigo and Jacoby^1,2,3^, as well as Mann *et al.*^4,5^, temperature reconstructions of the Common Era have become a key component of climate assessments^6,7,8,9^. Such reconstructions depend strongly on the composition of the underlying network of climate proxies^10^, and it is therefore critical for the climate community to have access to a community-vetted, quality-controlled database of temperature-sensitive records stored in a self-describing format. The Past Global Changes (PAGES) 2k consortium, a self-organized, international group of experts, recently assembled such a database, and used it to reconstruct surface temperature over continental-scale regions^11^(hereafter, ‘PAGES2k-2013’).

This data descriptor presents version 2.0.0 of the PAGES2k proxy temperature database (Data Citation 1). It augments the PAGES2k-2013 collection of terrestrial records with marine records assembled by the Ocean2k working group at centennial^12^ and annual^13^ time scales. In addition to these previously published data compilations, this version includes substantially more records, extensive new metadata, and validation. Furthermore, the selection criteria for records included in this version are applied more uniformly and transparently across regions, resulting in a more cohesive data product.

This data descriptor describes the contents of the database, the criteria for inclusion, and quantifies the relation of each record with instrumental temperature. In addition, the paleotemperature time series are summarized as composites to highlight the most salient decadal- to centennial-scale behaviour of the dataset and check mutual consistency between paleoclimate archives. We provide extensive Matlab code to probe the database-processing, filtering and aggregating it in various ways to investigate temperature variability over the Common Era. The unique approach to data stewardship and code-sharing employed here is designed to enable an unprecedented scale of investigation of the temperature history of the Common Era, by the scientific community and citizen-scientists alike.

## Methods

### Collaborative model

The database is the product of a community-wide effort, coordinated by PAGES through a network of nine working groups (http://www.pastglobalchanges.org). Calls for participation were disseminated broadly and regional leaders solicited input from scientists with relevant datasets and/or expertise. A provisional database was compiled into a uniform framework, then redistributed to regional groups for quality control and further additions. For this purpose, quality control plots, including basic metadata for each record, were prepared to enable coauthors of this data descriptor to efficiently recognize and correct errors. Examples of these plots are given in the Quality Control section, and the full collection is archived as pdf files in Data Citation 1.

### Data aggregation

The PAGES2k community aimed to identify records that are most relevant to understanding temperature evolution over the last 2000 years, while also assembling a uniform global database that can be culled to address a wide range of research questions. Specific criteria were developed to gather all published proxy records that meet five objective and reproducible criteria:

### Thermal sensitivity

Proxy records were gathered from archive types for which previous understanding of the proxy system indicated that the records are temperature-sensitive. Records were only included when the original study described the relation between the proxy value and one or more climate variables, including temperature, or when the correlation with nearby instrumental temperature data was high enough to reject the null hypothesis of zero correlation at the 5% level, taking into account both temporal autocorrelation and test multiplicity. Indeed, temporal autocorrelation is well known to reduce the number of degrees of freedom available to reject a null hypothesis^14^. The test multiplicity problem (aka the ‘multiple comparisons problem’ or ‘look-elsewhere effect’) is the propensity for false positives to arise when multiple hypothesis tests are conducted simultaneously; in this case, testing the null hypothesis at 1,000 grid points with a 5% level would be expected to yield fifty spurious ‘discoveries’^15^ even in the absence of any link to temperature. Our analysis controls for both effects (see ‘Relationship to temperature’).

In addition, regional and proxy experts who are authors on this data descriptor certified that the records reflect temperature variability and that they meet all other stated criteria (Supplementary Table 1). Note that temperature sensitivity does not preclude the potential for many proxy systems to be secondarily or additionally sensitive to other environmental variables, such as moisture availability.

### Record duration

A primary goal of the PAGES2k project is to understand climate dynamics over the entire Common Era. Records of this duration are most commonly accessible from sedimentary sequences that lack annual resolution; a minimum length of 500 years for these records serves as a coarse initial screen. For annually-banded terrestrial records (e.g., varves, glacier ice, tree rings), shorter-duration records that overlap with the instrumental period are important for calibration-validation exercises and for bridging between annually-resolved and lower-resolution records; as a result, annually resolved records from terrestrial archives over 300 years long were also included. Annually resolved records from marine archives (corals, molluscs) are rarely this long, but provide critical information where instrumental data are often sparse or absent, and were included in the database if they exceeded 50 years in duration.

### Chronological accuracy

Most records in this database are layer-counted, with a dating uncertainty of a few percent or less, but generally extend back less than 500 years. Other proxy records may span many millennia but some have chronologies that are too uncertain for centennial and finer-scale paleotemperature reconstructions. Recognizing, however, that lake and marine sediments accumulate at approximately constant rates, and considering the goal of building a comprehensive database from which records can be culled as necessary, depending on the scientific question, the initial screen for chronological control was relatively coarse. Once suitable records have been identified, their age-model uncertainty can be quantified using existing statistical procedures^16,17,18,19,20^, providing a useful basis for including or weighting individual records in paleotemperature reconstructions. Namely, when annual layers cannot be counted, the timelines for records selected for this database were constrained by at least one chronological control point near the most recent end of the record and another near the oldest part of the record, or 1 CE, whichever is younger. Records that are longer than 1,000 years must include at least one additional age approximately midway between the other two. What constitutes ‘approximately’ was open to reasonable interpretation but was typically within two centuries of the mid-point.

### Record resolution

PAGES2k scientific questions focus on centennial and finer time scales. Terrestrial and lacustrine records were included with average sample resolution of 50 years or finer. However, such records are rare from marine sediments, and thus a minimum average sample resolution of 200 years was accepted for this database. We also included 4 borehole records, although quantifying median resolution is less straightforward in boreholes than in other archives. The borehole records in the database are appropriate for examining decadal to multi-centennial scale variability, depending on the timeframe of interest^21^.

### Public availability

Proxy records used in the PAGES2k synthesis products are publicly available through previous publications or online data archives, or because their owners made them available for inclusion in this open-access data product. The original data for 49 records are made available for the first time in this data product (specified in Supplementary Table 1). Open access is a critical component of this endeavor, and led us to reject some records that would have been suitable under the other criteria. The focus on annual- to centennial-scale temperature of the past 2000 years led to the exclusion of those paleoclimate records that did not meet the resolution or geochronological control criteria required for meaningful inferences of the temperature history of Common Era.

### Relation to previous release

The selection criteria for this dataset are specific to the type of proxy archive; for some proxy types, the standards in this version were broadened compared to the criteria used previously by PAGES2k regional groups. In most regions, records have been added that have become available since the publication of PAGES2k-2013, or that were not used in the continental-scale reconstructions because they are not annually resolved and therefore did not conform to the reconstruction method used by a particular regional group. In Antarctica, for example, PAGES2k-2013 included only the longest annually resolved ice cores, whereas the present version includes shorter and decadal-scale-resolution records.

For other proxy types, more stringent criteria resulted in the exclusion of some records. The excluded records are tracked in Supplementary Table 2. In most regions, some records were excluded because they did not meet the stricter standards for the minimum length or temporal resolution (criteria detailed above), or because of ambiguities related to the temperature sensitivity of the proxy, or because they have been superseded by higher-quality records from the same site. Of the 641 records that together comprise the previously published PAGES2k datasets, 177 are now excluded, of which 124 are tree-ring-width series that are inversely related to temperature. To be included in the current database, tree-ring data were required to correlate positively (*P*<0.05) with local or regional temperature (averaged over the entire year or over the growing season). Trees whose growth increases with temperature (e.g., direct effect of temperature on physiological processes and photosynthetic rates) are more likely to produce a reliable expression of past temperature variability compared to trees that respond inversely to temperature, for which the proximal control on growth is moisture stress (e.g., evapotranspiration demand)^22^. Because many trees are more strongly influenced by moisture availability than by growing season temperatures^23^, including only the positive responders reduces the overall number of tree-ring records to a more selective subset (see Supplementary Information, section 1).

### Metadata

The current database includes a large number of metadata fields to facilitate the intelligent reuse of the data. Table 1 (available online only) lists a subset of information in a single-page format. Supplementary Table 1 includes additional metadata fields with critical information to convey the appropriate use of each dataset, namely: the PAGES2k identifier assigned for this data product, the identifier used in previous PAGES2k products by the Ocean2k working group^12,13^, or by PAGES2k-2013, whether the record is superseded by another in this version, the archive type, the primary publication citation, its associated digital object identifier (DOI; if one exists), the secondary publication citation and DOI, the URL link to where the data were archived by the original author, the associated data citation, the geographic coordinates (latitude, longitude, elevation), the name of the site, the ISO 3166-1 standard name of the country/ocean basin where it is located, the earliest and latest years covered by the record, the resolution of the time series (median spacing between consecutive observations), the type of proxy observation, the name of the variable used as the temperature-sensitive time series and its units, the physical feature whose temperature is sensed by the proxy (e.g., surface air temperature, sea-surface temperature), the part of the seasonal cycle recorded by the proxy, the direction of the relationship between the proxy and temperature (positive or negative), quality control (QC) comments, initials of PAGES2k Consortium author who performed QC certification, and a permalink to the dataset’s page at the NCEI-Paleo/World Data Service for Paleoclimatology.

### Annualization

Annualization is necessary to compare proxies of varying sampling resolution with instrumental observations or with each other. Records with a superannual resolution were interpolated to annual resolution via nearest-neighbor interpolation. Interpolating records may alter their spectral content^24^, but permits comparison of information on a common time grid and for shared spectral resolutions.

Seasonally-resolved proxies (e.g., most corals) were averaged to produce annual (ANN: Jan-Dec), DJF (December January February) and JJA (June July August) anomalies. Some records from glacier ice have a sampling resolution finer than 1 year. However, firn diffusion smooths subannual signals, such that the shortest recoverable periodicity is generally no shorter than 1 year^25,26^. Such records were therefore annualized to a Jan-Dec window.

The vast majority of records in the database are annually-resolved, and are not affected by this processing. We note, however, that many such records subsample part of the annual cycle (e.g., for tree rings, the growing season). For this purpose it is instructive to compare such records to annual (Jan-Dec), DJF and JJA averages of the HadCRUT4.2 temperature field (Technical Validation). The annualized data are archived alongside the original data, so either may be used in subsequent analyses.

### Code availability

The Matlab code (https://www.mathworks.com/products/matlab.html) necessary to reproduce the figures of this descriptor is available at https://github.com/CommonClimate/ PAGES2k_phase2 under a free BSD license.

## Data Records

### Proxy dataset

**Figure 1:**
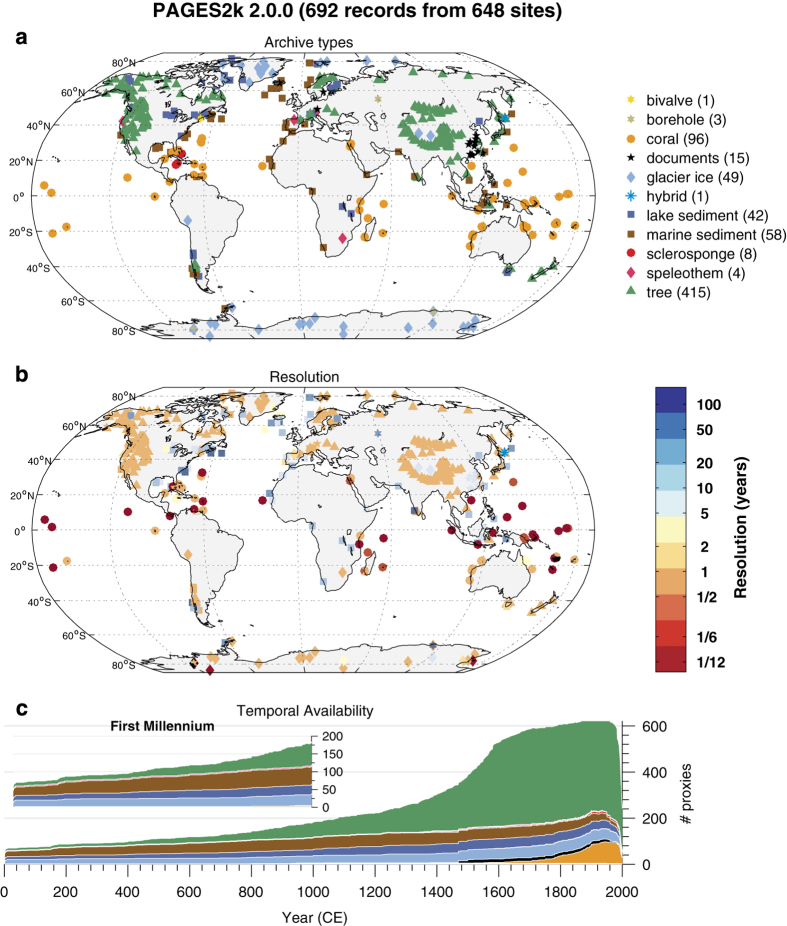
Spatiotemporal data availability in the PAGES2k database. (**a**) Geographical distribution, by archive type, coded by color and shape. (**b**) Temporal resolution in the PAGES2k database, defined here as the median of the spacing between consecutive observations. Shapes as in (**a**), colors encode the resolution in years (see colorbar). (**c**) Temporal availability, coded by color as in (**a**).

The PAGES2k temperature database (Fig. 1, Supplementary Fig. 1) includes 692 records (Data Citation 2 to Data Citation 477) from 49 countries and 11 distinct types of archives: 415 from trees (ring width and density), 96 from corals (e.g., isotopes, elemental composition, calcification rate), 58 from marine sediments (e.g., geochemistry, floral and faunal assemblages), 49 from glacier ice, 42 from lake sediments (e.g., floral and faunal assemblages, sediment accumulation, geochemistry), 15 from documentary sources, 8 from sclerosponges, 4 from speleothems, 3 from boreholes, 1 from bivalves, and 1 hybrid (tree/borehole) record. Each of these archives bears the imprint of a proxy system responding to temperature changes, with the signal recorded in one or more of the archive’s chemical, physical, or biological properties^27^. The details behind the collection, analysis and interpretation of each of the records in the database are beyond the scope of this data descriptor, and we refer readers to the original publications for that information.

The records cover a wide range of time spans, from a minimum of 52 years to a maximum of 2000 years. The average length is 760 years, the median 547 years, not counting the duration of any record beyond 2000 years; temporal resolution ranges from biweekly to centennial, with a majority of annual records. As seen in Fig. 1, many proxy records spanning the last 2000 years are not annually resolved, and in some regions, most of the available records of any length lack annual resolution. The mean resolution of non-tree archives is 11 years, the median 1 year. For sedimentary archives the mean and median resolutions are 25 and 18 years, respectively. A list of sites comprising the database, along with basic metadata, is presented in Table 1 (available online only), an expanded version of which is in Supplementary Table 1. Note that some sites include more than one proxy temperature record.

The majority (59%) of the records are based on tree rings because they are annually resolved, precisely dated, and geographically widespread, especially in the mid-latitudes of the Northern Hemisphere (Supplementary Information, section 1). The PAGES2k collection is unique among previous efforts in the amount of paleoclimate evidence from sources other than tree rings, such as lake and marine sediments, corals, glacier ice and speleothems, thus expanding the geographic and temporal coverage of the database, as well as mitigating potential issues regarding the use of tree rings for temperature reconstructions^28^.

While the vast majority of the records gathered herein were layer-counted, there are 87 sediment (marine or lake) datasets whose chronologies are derived from radiometric methods. For 41 of those datasets (47%), Data Citation 1 includes the primary geochronological information needed for a formal treatment of time uncertainty using various age-modelling techniques^19,29,30^. Additionally, 30 records (overlapping, but not exclusively, with the 41 above) include chronology ensembles from the Arc2k 1.1.1 dataset^31^. These include both sedimentary records with age ensembles derived via BACON^19^, and ice and varved records with age ensembles derived via BAM^32^.

**Figure 2:**
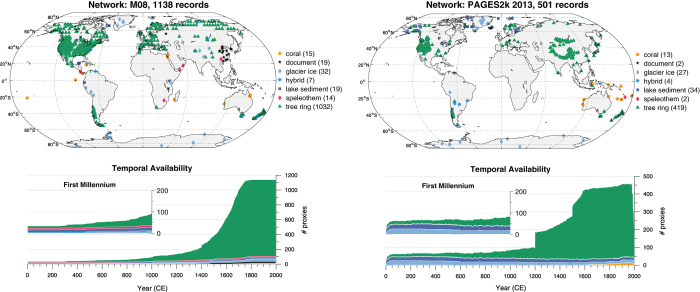
Data availability in the M08 (refs 33,34) and PAGES2k-2013 (ref. 11) databases. Graphical conventions as in Fig. 1. Note that the *y*-axis scale varies between plots on account of the large differences in number of records, but the first millennium inset uses the same scale between all comparable panels in Fig. 1 and this one, to highlight progress made in bolstering coverage of this time period.

For comparison, Fig. 2 displays the spatiotemporal distribution of proxy archives in the databases of Mann *et al.*^33,34^ (hereafter M08) and PAGES2k-2013 (ref. 11). While the M08 database contains 75% more records than this collection, these records are overwhelmingly land-based, from the Northern Hemisphere, and relatively short. Indeed, the M08 database is disproportionately composed of tree rings from North America, many of which start after 1000 CE, so that fewer than 100 records reach beyond this date. In contrast, the present collection contains 176 records out to 1000 CE, most of which are not tree-based. While the PAGES2k-2013 effort had succeeded in diversifying the network prior to 1,000 CE, it focused on terrestrial sites, and was dominated by tree-based records after 1200 CE. The proportion of records from the Southern Hemisphere is comparable between all three databases (15% in M08, 12% in PAGES2k-2013, 16% in this study), but the number of records from Antarctica has steadily improved between databases (8 in M08, 9 in PAGES2k-2013, 26 here). The present dataset therefore constitutes a major leap in terms of the diversity and duration of records, as well as oceanic and polar coverage. The present compilation also marks an unprecedented effort at rigorously assessing their quality as temperature indicators (Technical Validation). While the overall quantity of records has declined with respect to M08, this is largely the result of more selective inclusion criteria (Methods).

Indeed, a unique aspect of the PAGES2k effort is the richness of the metadata annotating each record. In Supplementary Table 1, all proxy records are accompanied by information about their paleotemperature interpretation, including where the proxy senses temperature (e.g., surface-air temperature, sea-surface temperature), the sign of this relationship (positive or negative), and the part of the annual cycle that is preferentially recorded (e.g., May June July). Some of the records from marine sediments were processed for additional quality control as described by ref. 12. The ‘QC Notes’ column of Supplementary Table 1 specifies data processing that was done, and explains modifications relative to the original data citation. In addition to the metadata in Supplementary Table 1, which are complete for every record, Data Citation 1 includes additional metadata for some records. The type of additional information depends on the proxy record and some of the information is missing for some records. For example, when available, the basis for the temperature interpretation is stated (e.g., calibration or first principles). Some records that were calibrated to temperature (e.g., ref. 35) include the native data from which the temperature series was derived, as well as a description of the calibration (equation, reference, uncertainty, units). This metadata structure follows the Linked Paleo Data (LiPD) structure, and the interested reader is referred to the associated publication^36^ for a full exposition of the format.

Accordingly, the database is primarily encoded as LiPD^36^ files: a structured, machine-readable format for paleoclimate data based on Javascript Object Notation (JSON) that accommodates the wide diversity of information comprising this database (PAGES2k_v2.0.0_LiPD.zip, Data Citation 1). Serializations are also available in the Python (PAGES2k_v2.0.0-ts.pklz, Data Citation 1), Matlab (PAGES2k_v2.0.0.mat, Data Citation 1) and R (PAGES2k_v2.0.0.Rdata, Data Citation 1) languages. Utilities for interacting with LiPD files in Matlab and Python are available at http://github.com/nickmckay/LiPD-utilities. Utilities in R are forthcoming.

### Instrumental temperature dataset

The ability of the proxy network to capture temperature information is assessed with respect to the instrumental HadCRUT4.2 dataset^37^, covering CE 1850–2014. The dataset merges surface air temperature over land (CRUTEM4) and sea-surface temperature over ocean regions (HadSST3). We use the Cowtan & Way version^38^ of the dataset, which corrects for missing values and incomplete post-1979 Arctic coverage via the use of satellite observations. Even with the correction, the HadCRUT4.2 dataset is incomplete, with about 60% of the monthly values missing, so the remaining missing values were infilled via the GraphEM^39^ algorithm. The graph was chosen via the graphical lasso^40^ using a sparsity parameter of 0.7%, which was chosen by cross-validation as the minimizer of the expected prediction error (HADCRUT4_median_GraphEM.mat, Data Citation 1).

**Figure 3:**
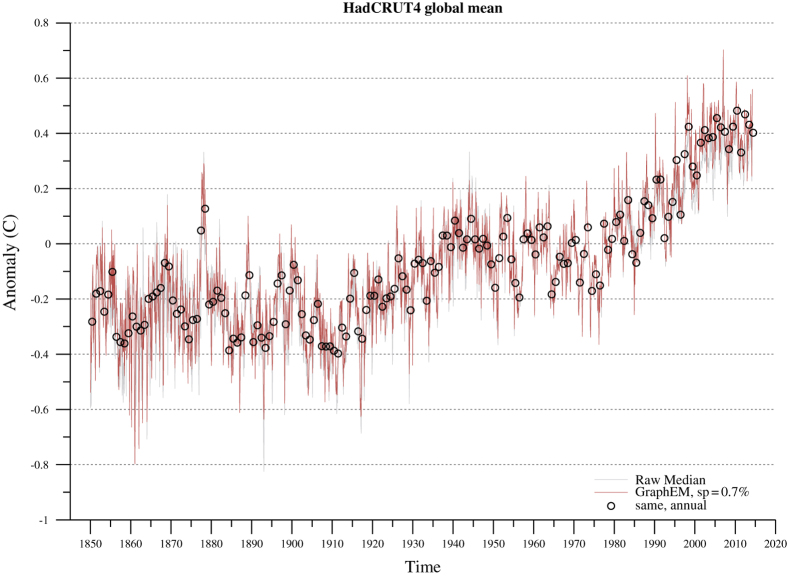
Global mean temperature from the HadCRUT4.2 dataset before (gray) and after (red) imputing missing monthly values via GraphEM. Black circles mark the yearly averages (mean annual temperature, or MAT) of GraphEM-imputed temperature values (red line).

The global (area-weighted) mean from this dataset is charted in Fig. 3. We note that this dataset may result in temperature variations whose amplitude is biased downwards in regions of poor observational coverage, hence potentially distorting proxy-temperature correlations. Regionally-specific temperature datasets (e.g., ref. 41 for Antarctica) would therefore be more appropriate in regional applications.

## Technical Validation

A unique challenge for technical validation of paleoclimatological datasets is that the target, here, site-local temperature over the Common Era, is unknown. Addressing this issue is an important objective of the current study. Our approach to validation includes comparison with the instrumental data for annually-resolved records, subsampling the dataset to assess reproducibility among proxy types and other subsets based on different screening criteria, and coarse-graining the time series to different extents to address issues related to combining records of different resolution and age certainty. Evidence that the records in the database reflect past temperature variability can be found in the original publications associated with each record. In addition, each series incorporated in the dataset was examined by one or more regional experts, who certified that each proxy record included in the database was accurate and related to temperature (Supplementary Table 1). This level of expert elicitation is unique among existing paleoclimate syntheses covering the last two millennia, and is a key value proposition of the PAGES2k crowd-curation process.

### Quality control

To facilitate quality control of individual records within the database, dashboards displaying raw data, their annualized version and the extent to which they may be informative of annual, JJA or DJF temperature were created. These figures are grouped by region or globally and included on the FigShare repository associated with this publication (Global_QCfig_bundle.pdf, Data Citation 1).

Fundamentally there are two ways to infer past temperatures from paleoclimate records. They can be calibrated using either:

1. direct (in-time) calibration; or:
2. indirect (space for time) calibration.

In the first approach, the record must overlap with the instrumental period (here: 1850–2014), and this period of overlap must contain enough points for a statistical calibration to an instrumental temperature product such as HadCRUT4 to be meaningful. In the second approach, one often uses transfer functions or laboratory-based culture experiments. Accordingly, summary plots for all records are divided into two categories, described below. The instrumental overlap threshold requirement is set at *n*=20 based on sensitivity tests (not shown). This parameter may be changed in the code associated with this dataset (see Code Availability).

### Records that can be calibrated in time

**Figure 4:**
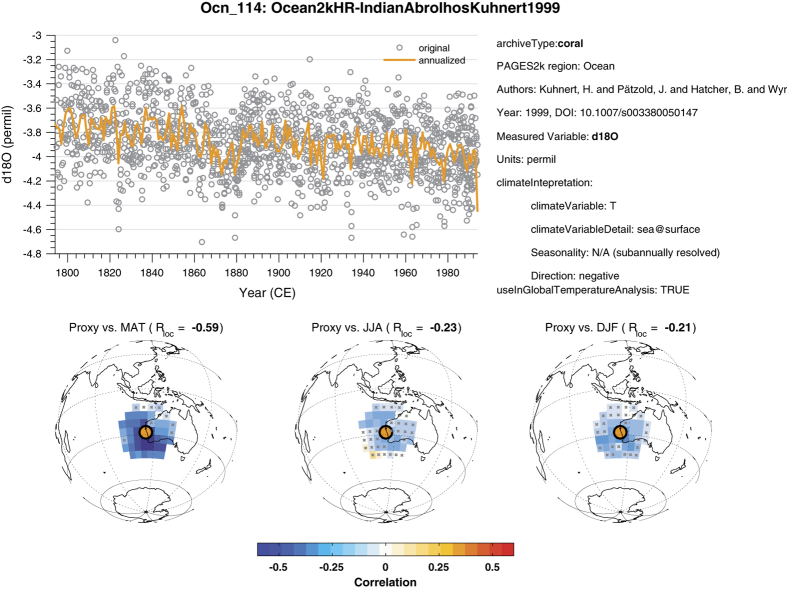
Quality-control plot for record Ocn_114 (Ocean_QCfig_bundle.pdf, Data Citation 1). See text for details as an example of record that can be calibrated in time.

Record Ocn_114 (ref. 42) (Fig. 4) is one such example. The top panel shows the (monthly) raw data as gray circles and an annualized curve whose color code matches that of Fig. 1a. The three bottom plots depict correlations with temperature grid points taken within a 2,000 km radius. The bottom left plot shows correlations with mean annual temperature (MAT), with insignificant correlations (as per an isospectral test^43^, 1,000 surrogates, 5% level) denoted by hatching. The local correlation is −0.59 and its bold font weight indicates statistical significance, also at the 5% level. Similar plots are shown for boreal summer (JJA, center) and boreal winter (DJF, right). Essential textual metadata are displayed on the right hand side. Similar plots follow identical conventions.

### Records that cannot be calibrated in time

**Figure 5:**
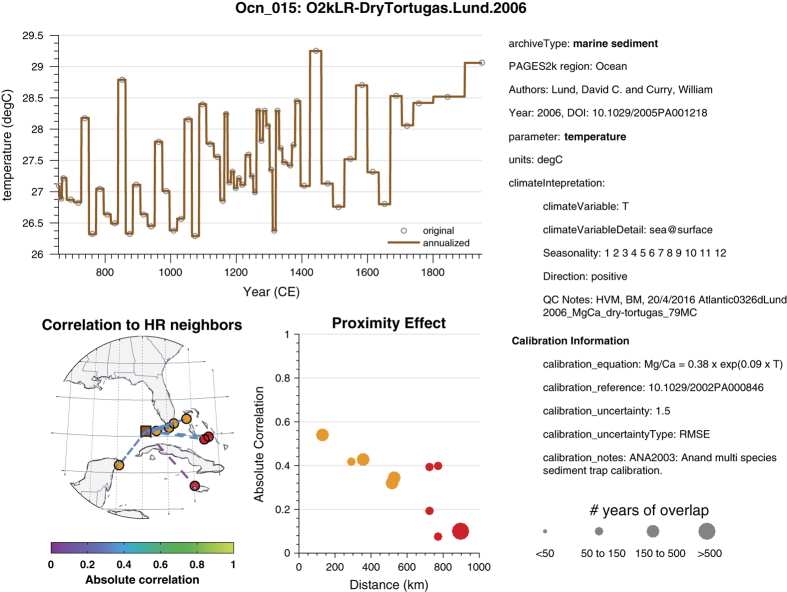
Quality-control plot for record Ocn_015 (Ocean_QCfig_bundle.pdf, Data Citation 1). See text for details as an example of record that cannot be calibrated in time.

If a record has too coarse a resolution, or ends too early, to contain 20 points over the 1850–2000 interval, it belongs to this category. One such record is Ocn_015, a foraminifera Mg/Ca record from the Caribbean^35^ (Fig. 5). This record was independently calibrated to temperature, as reflected in the ordinate of the time series plot (top left). As before, the right side of the page displays metadata, including the calibration method and its reported uncertainties. Since a comparison to an instrumental temperature series is neither possible nor meaningful for such a record, the bottom left panel displays its correlation to the 10 nearest high-resolution records (bottom left), coded by lines whose color represents the absolute correlation. Thick, solid lines represent significant correlations (again, as per ref. 43 at the 5% level), and thinner dashed lines represent correlations that did not pass the test. The bottom central panel stratifies these correlations by distance; the color corresponds to the proxy code (i.e., corals in orange, sclerosponges in red, c.f. Fig. 1a), with significant correlations circled in black. The size of the symbol scales with the number of years of overlap, as shown at the bottom right.

### Relationship to temperature

Here we examine the extent to which the database as a whole captures the observed temperature variability at local and regional scales. We do so via correlation analysis, which makes the common assumption that the relation between the proxy value and temperature over the twentieth century is representative of the entire record (stationarity). Unstable or multivariate associations between proxies and local temperature would represent a significant challenge to this assumption; however, this problem is not unique to paleoclimatology within the Earth sciences. The approach also assumes that the observational temperature time series itself is accurate and unbiased for each proxy site, which may not be true in areas of sparse coverage or complex topography.

The relationship between the current proxy database and the global temperature field is quantified via Pearson’s linear correlation coefficient (R) between proxy values and temperature averages (ANN, JJA, DJF). Statistical significance is established via a non-parametric, isospectral test^43^, which accounts for the loss of degrees of freedom due to large serial correlations common to proxy time series. Again, we restricted correlation analyses to records comprising a minimum of 20 samples over the instrumental era (CE 1850–2014), which limits the pool of proxies that may be evaluated in this way (*n*=597).

#### Regional screening

**Figure 6:**
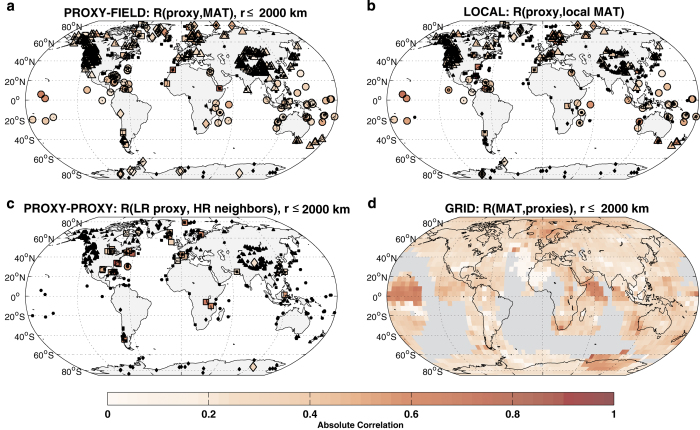
Relationships with temperature and other records. Median absolute correlations (**a**) of each record with mean annual temperature within a 2,000 km radius; (**b**) of each record with mean annual temperature at the nearest grid point; (**c**) of each low-resolution record with its 10 closest high-resolution proxy neighbors; (**d**) of temperature at each grid point and its proxy neighbors within a 2,000 km radius. Proxy-centric correlations (**a**–**c**) are reported in color if significant; as small black symbols if insignificant or not applicable. Grid-centric correlations (**d**) are reported in color if significant; in grey if insignificant or not computable (i.e., no proxy neighbor within 2,000 km).

First, we search for significant correlations (*P*<0.05) within a search radius *r*_*s*_, ensuring that correlations are local to regional. Compared to a global search, this limits the extent to which spurious correlations may arise, for instance, due to strong trends. Since spatial correlations are non-uniform and highly anisotropic, using a distance-based criterion that is uniform over the globe represents an oversimplification. No single distance is likely to be globally optimal, so its choice reflects a compromise between various factors: autocorrelation in land versus ocean temperatures, annual versus longer resolution, or seasonal biases. With *r*_*s*_=2,000 km^44^, 411 records show significant correlations with annual temperature—their absolute values are shown in Fig. 6a and their locations are shown in Supplementary Fig. S3. Results change modestly depending on the value used for *r*_*s*_.

#### Regional screening adjusted for the false discovery rate

Searching for potentially hundreds of suitable correlations within such a search radius runs the risk of false discoveries^45^. The problem of multiple hypothesis tests has long been known to statisticians and several solutions exist^46^. We use a method based on the false-discovery rate (FDR)^47^, adapted to the climate context^48,49^. In all, 277 records passed this test with annual data (Supplementary Fig. 4).

#### Local screening

The search may be further restricted to the nearest HadCRUT4.2 grid point. The results of this evaluation are mapped in Fig. 6b. In some cases this may be problematic because sites may sit at the boundary between grid cells. For sites located in the vicinity of frontal zones with large spatial temperature gradients, choosing the most appropriate neighbor can be particularly difficult. Gridded temperature data may represent observations from a range of elevations or environments, and therefore may not be representative of the archive’s actual location. Furthermore, the nearest grid point can in some instances be located thousands of kilometers from a site, because of the incomplete coverage of HadCRUT4. This limitation is particularly acute for Antarctic records, because of poor instrumental coverage over the Southern Ocean and Antarctic continent. A total of 181 records passed this test with annual data, including 5 from Antarctica, versus 9 in the regional+FDR case (Supplementary Fig. 5).

### Seasonal effects

The extent to which proxies are informative of annual temperature depends, sometimes very strongly, on the portion of the annual cycle which they preferentially sample^7^. Thus, before using seasonally-dependent proxies to reconstruct mean-annual temperature, one must ascertain the relationship between seasonal averages and the annual mean.

Supplementary Fig. S6 explores how much of the mean annual temperature (MAT) signal can be explained by boreal summer (JJA, top) versus winter (DJF, bottom) averages in the HadCRUT4.2 dataset. Correlations are generally very high (>0.8) in the tropics, where the MAT range is small, and low in the extra-tropics, particularly over northern hemisphere continental interiors for JJA, where the MAT range is large and dominated by winter synoptic variability. This means that proxies that preferentially record summer conditions may be adequate predictors of the annual mean if they are located in the tropics, but (all other things being equal) less so if they are located on Northern Hemisphere continents. Extratropical winter variability is known to dominate the annual average^44^, so correlations to the MAT primarily reflect winter conditions in those regions.

Table 2 summarizes the result of the aforementioned correlation-based screening for the three approaches (regional, regional with FDR control, and local), as well as the part of the year that goes into the annual average: ANN (calendar year), DJF, JJA, or April-March (AMA). The results make it clear that some proxy records are sensitive to JJA temperature, but not to DJF or annual temperature. The vast majority are tree-ring records from the Northern Hemisphere.

### Relationship to other proxies

A total of 95 proxies could not be directly correlated to MAT, either because they ended prior to 1850 or because they featured too few samples after this date. As an alternate validation method, we searched for significant correlations among the 10 closest neighbors from within the proxy dataset that can be correlated with HadCRUT4 (colored dots on Fig. 6a), and reported these significant correlations, along with their magnitude, in Fig. 6c. To minimize issues related to correlating time series with very different resolutions, the time series of proxy neighbors were smoothed to match the resolution of the target proxy. In regions where proxy-record density is high, this is a reasonable approach to assess the mutual consistency between various series; in sparsely sampled regions, this approach implies that proxy series that cannot be directly correlated to instrumental temperature must look like those that can, even if they belong to different climate settings. Moreover, despite the precautions taken with the isospectral test, correlations between a low-resolution record and its high-resolution neighbors are often driven by trends, even if no geophysical connection is present. Correlations between high- and low-resolution records must therefore be interpreted with caution. With these caveats in mind, 54 of the 95 proxies showed a significant correlation to neighboring high-resolution proxies.

### Grid-based spatial correlations

An alternate way to evaluate the extent to which variability in the global temperature field is captured by a heterogeneous network of paleoclimate proxies is to quantify the correlation between each grid cell’s instrumental temperature time series and that of all the proxy series within the radius *r*_*s*_^50^. Viewed in this manner, the statistical relationship between the regular grid and the irregular proxy network provides information about the extent to which different regions of the global temperature target field are represented by the paleoclimate data, and the strength of that relationship. The results of this evaluation are shown in Fig. 6d. It shows that surface temperature over 73% of the planet is significantly correlated to a proxy time series within a 2,000 km radius-about twice as great an area as covered by the previous PAGES2k compilation^11^.

### Global trends

Having quantified the degree to which proxy records from this dataset respond to temperature, we now synthesize the largest-scale thermal signal embedded therein. We do so by use of composite time series, which efficiently summarize the global trends captured in this large and diverse collection. Composites allow us to readily compare signals contained in various subsets of the database; these comparisons, in turn, are an essential check on the mutual consistency of the temperature proxies across regions, geographic settings, and proxy archives.

Our focus here is purposefully general, centered on multidecadal to centennial time scales and ignoring the spatial features. This simple approach is intended as a preliminary estimate of global mean temperature fluctuations over the past 2000 years, and sets the stage for future community endeavors. Indeed, several PAGES2k working groups are currently working to generate spatially resolved reconstructions of annual or seasonal temperature fluctuations at regional to global scales, as well as cross-validated estimates of global mean surface temperature using a variety of statistical approaches. The composites allow this database to be placed in the context of past reconstruction efforts, and to serve as a benchmark for future ones.

Following recent compilations^11–13^, we average all records (scaled to unit variance) into a composite. We do so at a coarse resolution by applying a simple binning procedure. Compositing makes two implicit assumptions:

- all proxy records are linearly related to global, mean-annual temperature.
- all proxy records are equally representative of global mean-annual temperature at any given time, and are thus given equal weight.

Given suitable transformations, (i) may be satisfied for a broad class of proxy records, even very nonlinear ones^51^. Assumption (ii) is more problematic, for three reasons. First, as Fig. 1a shows, the network is dominated by tree rings from the northern midlatitudes, whose temperature sensitivity is primarily linked to the growing season (boreal summer), representing only a fraction of the annual variance. Second, the mix of proxies is also non-stationary (Fig. 1c). Coral records are relatively abundant over the instrumental era but practically absent prior to 1,600 CE. Tree rings dominate the network after around 1,400 CE but less so prior to that. Finally, the majority of records have annual (or better) resolution, but some records have median resolution on the order of 100 years (Fig. 1b). The information density per unit time of such records is thus quite different. Furthermore, dating uncertainties in low-resolution (non layer-counted) proxies are not quantified, but are mitigated to some extent by multidecadal binning.

Despite assumptions (i) and (ii) above, and their potential violation, we suggest that a simple treatment of the data constitutes an informative appraisal of the largest-scale thermal signal embedded in the dataset. We emphasize, however, that the above concerns are all legitimate, and that more rigorous treatment of these assumptions should and will be applied in formal temperature reconstructions. Compositing involved the following processing steps:

#### Sign adjustment

Records were multiplied by −1 if their values decrease with increasing temperature (i.e., if their *interpDirection* parameter is negative); by +1 otherwise. This step ensures that all proxy values point upward (downward) in response to warming (cooling).

#### Normalization

Records were mapped to a standard normal distribution via inverse transform sampling^51^, resulting in zero mean and unit variance.

#### Binning

Since the main focus of this composite is on low-frequency (decadal and longer) variability, all records were averaged in bins of 25, 50, and 100 years. Binning also mitigates the effect of age uncertainties, as it is known that even small age offsets between annual records could otherwise cause large spurious trends in composites made from them^32^.

#### Scaling

Standardized composites were scaled to temperature over identical bins.

#### Screening

For high-resolution records (HR: median resolution finer than 5 years), we applied either no screening (*none*), regional temperature screening (*regional*), or regional screening adjusted for the false discovery rate (*regionalFDR*). For low-resolution records (LR: median resolution coarser than or equal to 5 years), *basicFilter* denotes records that comprise at least 20 values over the Common Era (Supplementary Fig. 2), while *hrNeighbors* denotes records with at least one significantly correlated HR neighbor (see above for the caveats of this approach).

#### Bootstrap

Uncertainties in the composite are quantified via a bootstrap approach^52^. This assumes exchangeability, and primarily measures sampling uncertainty. We plot 95% confidence intervals derived from an ensemble of 1,000 bootstrap samples; in general, such intervals widen with proxy attrition, as expected.

### Sensitivity analysis

**Figure 7:**
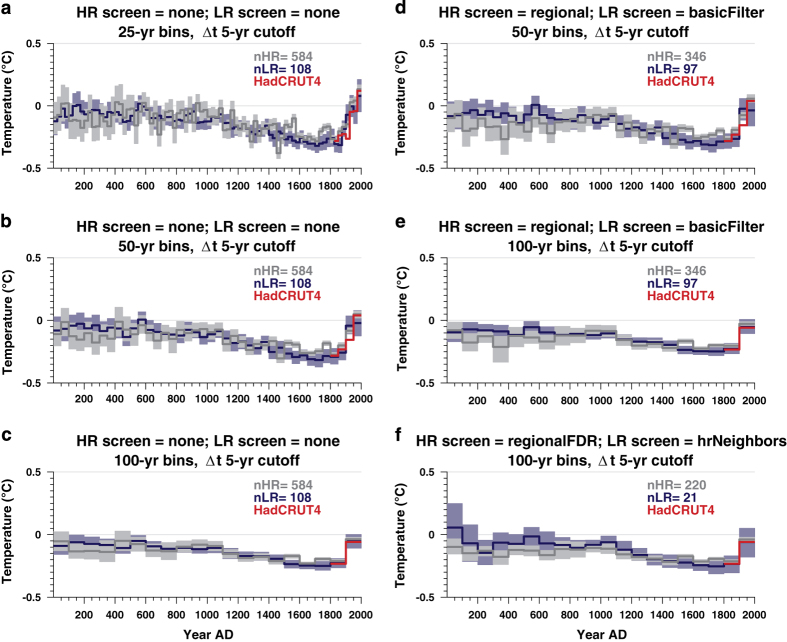
Global composites for various binning intervals and screening criteria, as indicated in subplot titles. The composites are scaled to temperature for comparison, and the shading denotes 95% bootstrap confidence intervals with 500 replicates, to constrain uncertainties. The cutoff between high-resolution (HR) and low-resolution (LR) records is defined as a median resolution of 5 years. Screening options comprise: no screening (*none*), regional temperature screening (*regional*), or regional screening adjusted for the false discovery rate (*regionalFDR*). For low-resolution records, *basicFilter* denotes records that comprise at least 20 values over the Common Era (Supplementary Fig. 2), while *hrNeighbors* denotes records with at least one significantly correlated HR neighbor.

Figure 7 presents the composites (HR in gray, LR in blue) and the HadCRUT4.2 target (red) scaled to temperature. Cases presented in the left column applied no screening, while the right column explores combinations of screening and binning interval. A striking feature is that in all cases, both HR and LR composites display a long-term cooling trend until the 19th century, after which an abrupt warming takes place, consistent with a very large body of literature^5,8,11,12^. We also note that temperature variability decreases with increasing bin size, as would be expected for data with random and independent errors.

We find the main results robust to screening choices, with the exception of the case in Fig. 7f (*regionalFDR, hrNeighbors*). The latter shows the most discrepancy between HR and LR, mainly because the number of LR proxies is very low (*n*=22) and they have little overlap with the instrumental era, making their temperature calibration unstable. In all cases the HR composites display slightly shallower variations than LR composites. There are two non-exclusive explanations for this. Firstly, it is known that some HR records, particularly the tree-ring chronologies that form the majority of this subset, can be limited in their ability to capture low-frequency variability beyond the mean segment length^53^. Second, LR records are known to redden climate signals, often exaggerating low-frequency variability at the expense of high frequencies^54^. Our analysis cannot distinguish between these two possibilities.

**Figure 8:**
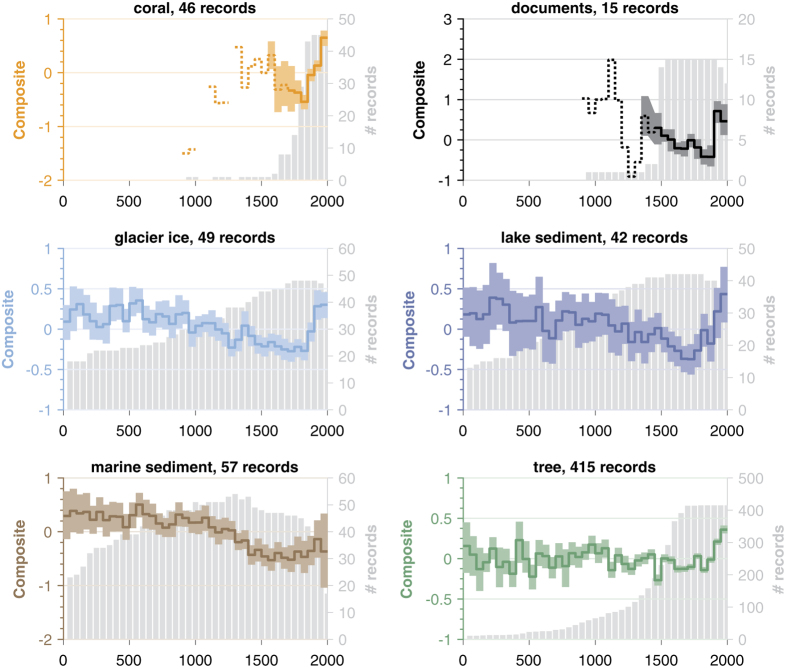
50-year binned composites stratified by archive type, for all types comprising 5 or more series. Composites with fewer than 10 available series are shown by a dotted curve, while solid lines indicate more than 10 series. Shading indicates 95% bootstrap confidence intervals with 500 replicates. Gray bars indicate the number of records per bin. The composites are expressed in standard deviation units, not scaled to temperature.

It is important to consider whether any of the primary features of the composite series are strongly controlled by a particular subset of proxies, or if they are shared among archive types. There are many potential ways to analyze this dataset. We give but one example in Fig. 8, gathering composites from individual archive types that include 5 or more records among the proxy collection: corals, documentary archives, glacier ice, lake and marine sediments, as well as trees. For this case we apply regional HR screening and basic LR filtering, then average records from coral, documentary, glacier ice, lake sedimentary, marine sedimentary, and tree-ring archives.

Most composites show a strong twentieth century warming trend that emerges above the variability of comparable centennial trends over the last two millennia. This is clearest in the tree- and coral-based composites, despite very large uncertainties in the latter during the seventeenth century, due to the paucity of records (Figs 1 and 8). An exception to this pattern is in the marine sediment composite (Fig. 8), which shows a cooling trend through most the Common Era. This may be explained by the low resolution of marine sediment records noted earlier, and the process of bioturbation of the sediment archive. These factors diffuse and damp changes occurring over years and decades (e.g., ref. 12), including the most recent warming. Local oceanographic factors may also play a role^12,55^.

Uncertainties in these composites include changes in sample size and available data network over time, the potential for non-climatic or non-temperature influences to bias these smaller subsets of the dataset, and the high spatial heterogeneity of subsample networks (Fig. 8). In general, uncertainty bands widen back in time (cf tree composite), with the notable exception of the marine sediment and documentary composites, which show widening bootstrap intervals in the last 2 bins, coincident with a drop in observational coverage in these archives. Note that multidecadal trends present in coral *δ*^18^O records from the eastern tropical Pacific may not be driven by temperature^13,56,57^ possibly biasing the trend of this coral composite. The network of lake records is regionally constrained (Fig. 1), and that composite may contain multiple environmental influences beyond temperature. As a result, from the lake subset only, we cannot exclude the possibility of above-modern levels of warmth in the third century CE, though uncertainty bands for early centuries are wide, and the recent rate of warming is clearly unprecedented over the Common Era.

The global composites derived from this dataset, despite their simplicity, supersede the composite-of-opportunity published in the last synthesis^11^, which was an average of regional indices obtained by very different means (hence not statistically homogeneous) and did not include the majority of the marine records gathered here. Nevertheless, the present composites share many similarities and some of the same caveats; namely, that a composite tends to give more weight to numerically abundant records (e.g., tree rings), and regions with more abundant observations (e.g., the Northern Hemisphere continents). An in-depth analysis of these composites, along with their climatic interpretation, will be the subject of a companion paper.

## Usage Notes

Data Citation 1 gathers data records in multiple digital formats, as well as quality control dashboards for all PAGES 2k regions (Table 3). This collection is the cornerstone of current and future efforts by the PAGES 2k Consortium to better reconstruct surface temperature, attribute its variability to climate forcings, understand its relationship to other components of the climate system, and constrain model simulations. It is appropriate for many purposes, ranging from developing reconstructions of climate indices (e.g., global mean surface temperature, NINO3.4) and fields, to proxy-proxy and proxy-model comparisons, and it was designed to be functional and relatively inclusive so that appropriate records could be selected, depending on the intended purpose, of which some are presently unforeseen.

The 692 temperature-sensitive records described and validated in this manuscript were selected based on the criteria listed above. In addition to these records, Data Citation 1 contains 2,240 ancillary time-series data from the same sites. Most (87%) are associated with tree-ring records from North America, including raw measurements, sample density and expressed population statistics; some are the native observations used to derive the temperature reconstructions included in the restricted group of 692 (e.g., Mg/Ca of foraminifera for sea-surface temperature); others are not directly related to climate but represent environmental changes at the site that might be useful in interpreting the climatic significance of the record (e.g., sedimentary magnetic susceptibility); some are proxy climate records that are sensitive to climate variables other than temperature. These 2,240 records are all timeseries, in that the datasets are year/data pairs. These ancillary time series are provided ‘as is’; the authors make no claims or guarantee as to their scientific usage.

Within Data Citation 1, the 692 temperature-sensitive records that comprise v2.0.0 are each assigned a unique PAGES2k identifier, as listed in Table 1 (available online only) and Supplementary Table 1. The 2,240 ancillary records are not assigned PAGES2k identifiers. In addition, the 692 records are easily discoverable in Data Citation 1 (PAGES2k_v2.0.0_LiPD.zip, PAGES2k_v2.0.0.mat, PAGES2k_v2.0.0.RData, PAGES2k_v2.0.0-ts.pklz) by querying the metadata property ‘paleoData_useInGlobalTemperatureAnalysis’, which is set to ‘TRUE’ only for the 692 temperature-sensitive records described here.

Several factors stand in the way of the PAGES2k compilation being fully comprehensive: records are continuously being generated and published, while some existing records are not publicly archived. This synthesis represents a major community effort to compile data records and captures a substantial majority of relevant records; it is to be continuously expanded and curated by the PAGES2k community. In addition to Data Citation 1, the entire database will be made available as part of a more comprehensive, web-based data management platform (http://linked.earth). This cyberinfrastructure facilitates crowd curation, transparent discussions of proxy interpretations, tracking and versioning of paleo data, and is supported by the first paleoclimate ontology (http://linked.earth/projects/ontology). In the near future, the current PAGES2k temperature dataset will be integrated with other paleoclimate datasets in this platform—for example, one dedicated to water isotopes (Iso2k^58^)—to enable the data-intensive studies of the last 2,000 years envisaged by the PAGES2k community^59^. The LinkedEarth cyberinfrastructure will enable crowdsourced additions and edits to the database, allowing it to be a living entity, with careful versioning to ensure workflow reproducibility.

Our versioning scheme is as follows: the version number for a data compilation is of the form *C*_1_.*C*_2_.*C*_3_, where *C*_1_ is a counter associated with a publication of the dataset (e.g., ref. 1), *C*_2_ is a counter updated every time a record is added or removed, and *C*_3_ is a counter updated every time a modification is made to the data or metadata in an individual record. The dataset published here is thus v2.0.0 of the PAGES2k proxy temperature dataset. Future versions of the dataset, along with a change log that specifies the modifications associated with each new version, will be posted at http://wiki.linked.earth/PAGES2k. This versioning applies only to the temperature-sensitive records in Data Citation 1; changes to ancillary time series are not tracked.

In addition, an archival version of the dataset is available on the website of NCEI-Paleo/World Data Service for Paleoclimatology (https://www.ncdc.noaa.gov/paleo/study/21171), in both the LiPD format and the WDS ASCII template format developed in conjunction with the PAGES2k consortium, that will be updated and versioned as the dataset continues to evolve.

## Additional Information

**How to cite this article:** PAGES2k Consortium. A global multiproxy database for temperature reconstructions of the Common Era. *Sci. Data* 4:170088 doi: 10.1038/sdata.2017.88 (2017).

**Publisher’s note:** Springer Nature remains neutral with regard to jurisdictional claims in published maps and institutional affiliations.

## Supplementary Material



Supplementary Information

Supplementary Tables

## Figures and Tables

**Table 2:** Number of records retained in the correlation-based screening depending on the method used and the part of the annual cycle selected.

**method**	**ANN**	**DJF**	**JJA**	**AMA**
reg	411	396	488	398
fdr	277	230	318	260
loc	181	114	228	168
ANN: Mean Annual Temperature. DJF: December-January-February. JJA: June July August. AMA: April-March (‘tropical year’) reg: regional screening. fdr: regional screening, controlling for a 5% false discovery rate. loc: local screening. See text for details.				

**Table 1:** Proxy series included in this synthesis

**PAGES ID**	**Site Name**	**Lat (°)**	**Long (°)**	**Archive**	**Proxy**	**Min Year (CE)**	**Max Year (CE)**	**Resolution (yr)**	**Reference**	**Data Citation**	**In Composites**
Afr_004	Lake Tanganyika	−6.03	28.53	lake sediment	TEX86	504	1986	21	^60^	2	yes
Afr_005	Lake Malawi	−10.0033	34.2883	lake sediment	TEX86	1283	1996	18	^61^	3	yes
Afr_012	Cold Air Cave	−24.02	29.11	speleothem	d18O	1635	1992	1	^62^	4	yes
Ant_001	Talos Dome	−72.8	159.06	glacier ice	dD	1232	1995	1	^63^	5	yes
Ant_002	DSS	−66.77	112.807	glacier ice	d18O	173	1995	1	^64^	6	yes
Ant_003	Plateau Remote	−84	43	glacier ice	d18O	2	1986	1	^11^	1	yes
Ant_004	Coastal DML	−70.86	11.54	glacier ice	d18O	1533	1994	1	^65^	1	yes
Ant_005	Site DML05	−75	−0.01	glacier ice	d18O	166	1996	1	^66^	7	yes
Ant_006	WDC05A	−79.46	−112.09	glacier ice	d18O	786	2005	1	^67^	8	yes
Ant_007	WDC06A	−79.46	−112.09	glacier ice	d18O	−50	2006	<1	^67^	8	yes
Ant_008	US-ITASE-2000-1	−79.3838	−111.24	glacier ice	d18O	1673	2001	<1	^67^	8	yes
Ant_010	James Ross Island	−64.2017	−57.685	glacier ice	dD	−1	2007	1	^68^	1	yes
Ant_011	Siple Station	−75.92	−84.25	glacier ice	d18O	1417	1983	1	^69^	1	yes
Ant_012	Berkner Island (South)	−79.57	−45.72	glacier ice	d18O	1000	1992	1	^70^	9	yes
Ant_013	Dome C	−75.1	123.39	glacier ice	dD	3	1919	14	^71^	10	yes
Ant_014	Dome C	−75.1	123.39	glacier ice	d18O	3	1919	14	^72^	11	yes
Ant_015	Dome F 2001	−77.32	39.7	glacier ice	d18O	695	1875	10	^73^	1	yes
Ant_016	Dome F 1993	−77.32	39.7	glacier ice	d18O	424	1467	4	^74^	1	yes
Ant_017	Ferrigno	−74.57	−86.9	glacier ice	dD	1703	2010	1	^75^	1	yes
Ant_018	DSS	−66.77	112.807	borehole	borehole	−6742	1995	102	^76^	1	no
Ant_019	MES	−77.515	167.6765	glacier ice	dD	1473	2007	<1	^77^	12	yes
Ant_020	Site DML07	−75.58	−3.43	glacier ice	d18O	1000	1994	1	^66^	13	yes
Ant_021	Site DML17	−75.17	6.5	glacier ice	d18O	1000	1997	1	^66^	14	yes
Ant_022	TALDICE	−72.82	159.18	glacier ice	d18O	4	1991	12	^78^	1	yes
Ant_023	Taylor Dome	−77.78	158.72	glacier ice	d18O	−25	1938	2	^79^	15	yes
Ant_024	US-ITASE-2002-4	−86.5	−107.99	glacier ice	d18O	1594	2003	<1	^67^	8	yes
Ant_025	VLG	−77.3302	162.5332	glacier ice	dD	1140	2000	1	^80^	16	yes
Ant_026	Vostok	−78.2785	104.8005	glacier ice	dD	1654	2010	1	^81^	1	yes
Ant_027	WAIS-Divide	−79.463	−112.125	borehole	borehole	8	2007	1	^21^	1	no
Ant_028	WDC06A	−79.46	−112.09	glacier ice	dD	−50	2006	<1	^67^	8	yes
Arc_001	Blue Lake	68.09	−150.47	lake sediment	varve thickness	730	2000	1	^82^	17	yes
Arc_002	Avam-Taimyr	72	101	tree	TRW	−1	2000	1	^83^	18	yes
Arc_004	Lower Murray Lake	81.35	−69.53	lake sediment	sed accumulation	−1	2000	1	^84^	19	yes
Arc_005	Camp Century	77.17	−61.13	glacier ice	d18O	1242	1967	1	^85^	20	yes
Arc_007	Gulf of Alaska	61.03	−146.59	tree	TRW	800	2010	1	^86^	21	yes
Arc_008	Yukon	67.9	−140.7	tree	TRW	1177	2000	1	^87^	22	yes
Arc_011	GISP2	72.1	−38.08	glacier ice	d18O	818	1987	1	^88^	23	yes
Arc_014	Lake Lehmilampi	63.62	29.1	lake sediment	varve thickness	1	1800	1	^89^	24	yes
Arc_016	Indigirka	69.5	147	tree	TRW	1259	1994	1	^90^	25	yes
Arc_018	Austfonna	79.83	24.02	glacier ice	d18O	1400	1998	1	^91^	26	yes
Arc_020	Lake C2	82.83	−77.9	lake sediment	varve thickness	−1	1987	1	^92^	27	yes
Arc_022	Hvítárvatn	64.6	−19.8	lake sediment	varve thickness	−1	2000	1	^93^	28	yes
Arc_024	Lena River	70.67	125.87	tree	TRW	1490	1994	1	^94^	29	yes
Arc_025	Lake Donard Baffin Island	66.73	−61.35	lake sediment	varve thickness	752	1992	1	^95^	30	yes
Arc_026	Lake Nataujärvi	61.81	24.68	lake sediment	varve property	−1	1800	1	^96^	31	yes
Arc_027	NGT B16	73.94	−37.63	glacier ice	d18O	1478	1992	1	^97^	32	yes
Arc_028	NGT B18	76.62	−36.4	glacier ice	d18O	871	1992	1	^97^	33	yes
Arc_029	NGT B21	80	−41.14	glacier ice	d18O	1397	1993	1	^97^	34	yes
Arc_030	Big Round Lake	69.87	−68.83	lake sediment	varve thickness	971	2000	1	^98^	35	yes
Arc_032	NGRIP1	75.1	−42.32	glacier ice	d18O	−1	1995	1	^99^	36	yes
Arc_033	Agassiz	80.7	−73.1	glacier ice	d18O	−1	1972	1	^100^	37	yes
Arc_034	Crete	71.12	−37.32	glacier ice	d18O	553	1973	1	^101^	38	yes
Arc_035	Dye	65.18	−43.83	glacier ice	d18O	1	1978	1	^101^	39	yes
Arc_036	GRIP	72.58	−37.64	glacier ice	d18O	1	1979	1	^101^	40	yes
Arc_037	Iceland	64.77	−18.37	documents	historic	945	1935	30	^102^	41	yes
Arc_040	Moose Lake	61.35	−143.6	lake sediment	midge	−718	1963	36	^103^	42	yes
Arc_041	Hudson Lake	61.9	−145.66	lake sediment	midge	−837	1997	47	^104^	43	yes
Arc_042	Screaming Lynx Lake	66.07	−145.4	lake sediment	midge	−1067	1988	51	^104^	43	yes
Arc_043	Braya Sø	67	−50.7	lake sediment	alkenone	−998	1999	29	^105^	44	yes
Arc_044	Devon Ice Cap	75.33	−82.5	glacier ice	d18O	1	1971	5	^106^	45	yes
Arc_045	Penny Ice Cap P96	67.25	−66.75	glacier ice	d18O	5	1980	25	^107^	46	yes
Arc_050	Lake Hampträsk	60.28	25.42	lake sediment	midge	1359	1994	14	^108^	47	yes
Arc_051	Lake Pieni-Kauro	64.28	30.12	lake sediment	chironomid	462	1979	44	^109^	47	yes
Arc_053	Penny Ice Cap P96	67.25	−65.75	glacier ice	melt	9	1984	25	^110^	48	yes
Arc_054	Lake 4	65.1	−83.79	lake sediment	chironomid	634	1997	50	^111^	49	yes
Arc_059	Renland	71.27	−26.73	glacier ice	d18O	3	1983	5	^100^	37	yes
Arc_061	Polar Urals	66.9	65.6	tree	TRW	891	2006	1	^112^	21	yes
Arc_062	Tornetrask	68.26	19.6	tree	MXD	−39	2010	1	^113^	50	yes
Arc_063	Jamtland	63.2475	13.3375	tree	MXD	783	2011	1	^114^	21	yes
Arc_064	Akademii Nauk Ice Cap, Severnaya Zemlya (Russian Arctic)	80.52	94.82	glacier ice	d18O	900	1998	1	^115^	51	yes
Arc_065	Arjeplog	66.3	18.2	tree	delta Density	1200	2010	1	^116^	1	yes
Arc_066	Armarnaes	65.9	16.1	tree	delta Density	1550	2010	1	^117^	1	yes
Arc_067	Hallet Lake	61.5	−146.2	lake sediment	BSi	116	2005	11	^118^	52	yes
Arc_068	Kittelfjall	65.2	15.5	tree	delta Density	1550	2007	1	^117^	1	yes
Arc_069	Kongressvatnet	78.0217	13.9311	lake sediment	alkenone	232	2008	10	^119^	53	yes
Arc_070	Lake E	67	−50.7	lake sediment	alkenone	−3642	1876	19	^120^	44	yes
Arc_071	Laanila	68.4917	27.3333	tree	MXD	800	2005	1	^121^	1	yes
Arc_072	Lomonosovfonna, Svalbard	78.8647	17.425	glacier ice	d18O	769	1997	1	^122^	1	yes
Arc_073	Mackenzie Delta	68.625	−133.87	tree	TRW	1245	2007	1	^123^	54	yes
Arc_074	Forfjorddalen	68.73	15.73	tree	MXD	1100	2007	1	^121^	1	yes
Arc_075	Prince-of-Wales, Ellesmere Isl.	78.4	−80.4	glacier ice	d18O	151	1995	1	^124^	1	yes
Arc_076	Soper Lake, Baffin Island, Canada	62.917	−69.883	lake sediment	varve thickness	1514	1992	1	^125^	55	yes
Arc_077	Tjeggelvas	66.6	17.6	tree	delta Density	1550	2010	1	^117^	1	yes
Arc_078	Windy Dome	80.783	65.63	glacier ice	d18O	1225	1995	1	^124^	1	yes
Arc_079	Yamalia	66.8	68	tree	TRW	914	2003	1	^126^	56	yes
Arc_080	Windy Dome	80.783	65.63	glacier ice	melt	1225	1995	1	^124^	1	yes
Asi_001	Altai Mt., Aktru Valley	50.08	87.77	tree	TRW	1495	1998	1	^127^	57	yes
Asi_002	Altai Mt., Djasator	49.62	88.1	tree	TRW	1561	2000	1	^128^	58	yes
Asi_003	Altai Mt., Jablonsky Pass.	50.87	85.23	tree	TRW	1568	1995	1	^129^	59	yes
Asi_004	Altai Mt., Kuraisky Ridge	50.3	87.83	tree	TRW	1538	2000	1	^128^	60	yes
Asi_005	Altai Mt., Kuraisky Steppe	50.27	87.83	tree	TRW	1497	2000	1	^128^	61	yes
Asi_006	Altai Mt., Samakha Steppe	49.72	87.28	tree	TRW	1542	2000	1	^128^	62	yes
Asi_007	Altai Mt., Korumdu Valley	50.14	87.72	tree	TRW	1418	1999	1	^127^	63	yes
Asi_008	Altai Mt., Tjute Valley	50.12	87.92	tree	TRW	1554	2000	1	^128^	64	yes
Asi_009	Altai Mt., Ust Ulagan Lake	50.48	87.67	tree	TRW	1550	1995	1	^127^	65	yes
Asi_010	Altai Mt., Ulagan Valley	50.68	87.97	tree	TRW	1555	2000	1	^128^	66	yes
Asi_011	Altai Mt., Ust Ulagan Lake	50.48	87.65	tree	TRW	1581	1994	1	^130^	67	yes
Asi_012	Altai Mt., Ust Ulagan Lake	50.48	87.65	tree	TRW	1581	1994	1	^130^	67	yes
Asi_013	Altai Mt., Ust Ulagan Lake	50.48	87.65	tree	TRW	1581	1994	1	^130^	67	yes
Asi_014	Altai Mt., Ust Ulagan Lake	50.48	87.65	tree	TRW	1581	1994	1	^130^	67	yes
Asi_015	Altai Mt., Ust Ulagan Lake	50.48	87.65	tree	TRW	1581	1994	1	^130^	67	yes
Asi_016	Altai Mt., Ust Ulagan Lake	50.48	87.65	tree	TRW	1581	1994	1	^130^	67	yes
Asi_017	Altai Mt., Ust Ulagan Lake	50.48	87.65	tree	TRW	1581	1994	1	^130^	67	yes
Asi_018	Altai Mt., Ust Koksa Hill	50.15	85.37	tree	TRW	1581	1994	1	^130^	68	yes
Asi_019	Altai Mt., Ust Koksa Hill	50.15	85.37	tree	TRW	1581	1994	1	^130^	68	yes
Asi_020	Altai Mt., Ust Koksa Hill	50.15	85.37	tree	TRW	1581	1994	1	^130^	68	yes
Asi_021	Altai Mt., Ust Koksa Hill	50.15	85.37	tree	TRW	1581	1994	1	^130^	68	yes
Asi_022	Altai Mt., Ust Koksa Hill	50.15	85.37	tree	TRW	1581	1994	1	^130^	68	yes
Asi_023	Altai Mt., Ust Koksa Hill	50.15	85.37	tree	TRW	1581	1994	1	^130^	68	yes
Asi_024	Altai Mt., Ust Koksa Hill	50.15	85.37	tree	TRW	1581	1994	1	^130^	68	yes
Asi_025	BT001	27.58	90.65	tree	TRW	1294	2003	1	^131^	69	yes
Asi_026	BT002	27.67	90.73	tree	TRW	1454	2003	1	^131^	70	yes
Asi_027	BT003	27.7	90.77	tree	TRW	1520	2003	1	^131^	71	yes
Asi_028	BT004	27.7	90.68	tree	TRW	1481	2003	1	^131^	72	yes
Asi_029	BHUTTD	27.67	90.72	tree	TRW	1450	2003	1	^132^	73	yes
Asi_030	BT006	27.63	90.13	tree	TRW	1400	2005	1	^131^	74	yes
Asi_031	BT008	27.58	90.65	tree	TRW	1280	2003	1	^131^	75	yes
Asi_032	BT009	27.42	90.97	tree	TRW	1457	2002	1	^131^	76	yes
Asi_033	BT010	27.25	89.38	tree	TRW	1484	2005	1	^131^	77	yes
Asi_034	BHUTSP	27.45	90	tree	TRW	1280	2005	1	^132^	73	yes
Asi_035	BT018	27.95	89.75	tree	TRW	1453	2006	1	^131^	78	yes
Asi_036	BT011	27.45	90.15	tree	TRW	1456	2005	1	^131^	79	yes
Asi_037	BT005	27.45	90.15	tree	TRW	1582	2006	1	^131^	80	yes
Asi_038	CHIN020	30.23	100.27	tree	TRW	1306	2007	1	^131^	81	yes
Asi_039	CHIN021	28.98	99.93	tree	TRW	1380	2007	1	^131^	82	yes
Asi_040	CHIN018	29.28	100.08	tree	TRW	1540	2006	1	^131^	83	yes
Asi_041	CHIN019	29.15	99.93	tree	TRW	1509	2006	1	^131^	84	yes
Asi_042	CHIN027	27.33	99.3	tree	TRW	1348	2007	1	^131^	85	yes
Asi_043	DEZQIN	34.75	100.82	tree	TRW	1287	2004	1	^132^	73	yes
Asi_044	GOUQIN	34.73	100.8	tree	TRW	1346	2004	1	^132^	73	yes
Asi_045	HEBQIN	34.73	100.78	tree	TRW	1475	2004	1	^132^	73	yes
Asi_046	LAJQIN	34.72	100.72	tree	TRW	1446	2004	1	^132^	73	yes
Asi_047	GHEGAN	37.93	101.53	tree	TRW	1288	2000	1	^132^	73	yes
Asi_048	CHIN006	36.3	98.08	tree	TRW	159	1993	1	^132^	86	yes
Asi_049	CHIN005	37	98.08	tree	TRW	840	1993	1	^132^	87	yes
Asi_050	DUSHJP	36.65	98.08	tree	TRW	840	1993	1	^132^	73	yes
Asi_051	MQAXJP	35.07	100.35	tree	TRW	1082	2001	1	^132^	73	yes
Asi_052	MQBXJP	34.78	99.78	tree	TRW	470	2002	1	^132^	73	yes
Asi_053	MQDXJP	34.72	99.67	tree	TRW	1163	2001	1	^132^	73	yes
Asi_054	MQFXJP	34.75	99.68	tree	TRW	1230	2002	1	^132^	73	yes
Asi_055	MQRXJP	34.75	99.68	tree	TRW	960	2002	1	^132^	73	yes
Asi_056	TDAXJP	34.78	100.8	tree	TRW	1340	2002	1	^132^	73	yes
Asi_057	TDBXJP	34.78	100.82	tree	TRW	1400	2002	1	^132^	73	yes
Asi_058	HBLXJP	34.78	100.82	tree	TRW	1520	2002	1	^132^	73	yes
Asi_059	HBMXJP	34.78	100.82	tree	TRW	1310	2002	1	^132^	73	yes
Asi_060	HBHXJP	34.78	100.82	tree	TRW	1500	2002	1	^132^	73	yes
Asi_061	BARELC	33.75	107.8	tree	TRW	1600	1992	1	^132^	73	yes
Asi_062	CHIN004	34.48	110.08	tree	TRW	1540	1990	1	^132^	88	yes
Asi_063	SANGTS	33.65	107.8	tree	TRW	1575	1992	1	^132^	73	yes
Asi_064	CHIN029	43.85	93.3	tree	TRW	1571	2002	1	^131^	89	yes
Asi_065	TIANMU	30.33	119.43	tree	TRW	1590	2007	1	^132^	73	yes
Asi_066	WULANJ	37.03	98.68	tree	TRW	150	2000	1	^132^	73	yes
Asi_067	QUMAJP	33.8	96.13	tree	TRW	1480	2002	1	^132^	73	yes
Asi_068	ZHIDJP	33.72	96.28	tree	TRW	1374	2002	1	^133^	90	yes
Asi_069	ZHANGX	34.63	104.47	tree	TRW	1568	2006	1	^131^	73	yes
Asi_070	CHIN017	28.9	99.75	tree	TRW	1452	2007	1	^131^	91	yes
Asi_071	CHIN016	31.78	101.92	tree	TRW	1575	2007	1	^131^	92	yes
Asi_072	PTCYUN	27.37	99.37	tree	TRW	1498	2007	1	^132^	73	yes
Asi_073	CHIN027	27.33	99.3	tree	TRW	1348	2007	1	^131^	85	yes
Asi_074	HXBURU	43.18	87.18	tree	TRW	1543	1993	1	^132^	73	yes
Asi_075	DQHZHO	35	100.07	tree	TRW	1433	2004	1	^132^	73	yes
Asi_076	CHIN026	27.62	99.8	tree	TRW	1516	2007	1	^131^	93	yes
Asi_077	HYGJUP	38.7	99.68	tree	TRW	540	2006	1	^132^	73	yes
Asi_078	MQACJP	35.07	100.35	tree	TRW	1082	2001	1	^132^	73	yes
Asi_079	CHIN037	27.58	99.35	tree	TRW	1429	2005	1	^132^	94	yes
Asi_080	CHIN038	27.58	99.28	tree	TRW	1542	2005	1	^132^	95	yes
Asi_081	CHIN039	28.03	99.02	tree	TRW	1489	2005	1	^132^	96	yes
Asi_082	CHIN040	28.03	98.98	tree	TRW	1393	2005	1	^132^	97	yes
Asi_083	CHIN041	27.88	98.4	tree	TRW	1530	2005	1	^132^	98	yes
Asi_084	CHIN050	37.47	97.23	tree	TRW	843	2001	1	^132^	99	yes
Asi_085	CHIN051	37.47	97.22	tree	TRW	828	2001	1	^132^	100	yes
Asi_086	CHIN052	37.45	97.53	tree	TRW	404	2002	1	^132^	101	yes
Asi_087	CHIN053	37.43	98.05	tree	TRW	451	2002	1	^132^	102	yes
Asi_088	CHIN054	37.45	97.78	tree	TRW	711	2003	1	^132^	103	yes
Asi_089	CHIN055	37.52	97.05	tree	TRW	1237	2002	1	^132^	104	yes
Asi_090	CHIN056	34.47	110.08	tree	TRW	1458	2005	1	^132^	105	yes
Asi_091	CHIN057	34.47	110.08	tree	TRW	1512	2005	1	^132^	106	yes
Asi_092	CHIN058	34.47	110.08	tree	TRW	1359	2005	1	^132^	107	yes
Asi_093	CHIN059	33.8	96.13	tree	TRW	1480	2002	1	^132^	108	yes
Asi_094	CHIN060	37.32	98.4	tree	TRW	943	2003	1	^132^	109	yes
Asi_095	CHIN061	37.03	98.63	tree	TRW	857	2003	1	^132^	110	yes
Asi_096	CHIN062	37.03	98.67	tree	TRW	845	2001	1	^132^	111	yes
Asi_097	CHIN063	36.75	98.22	tree	TRW	681	2001	1	^132^	112	yes
Asi_098	CHIN064	36.68	98.42	tree	TRW	900	2001	1	^132^	113	yes
Asi_099	CHIN065	32.67	95.72	tree	TRW	1290	2006	1	^132^	114	yes
Asi_100	CHIN066	33.72	96.28	tree	TRW	1374	2002	1	^132^	90	yes
Asi_101	INDO005	−5.5	122.8	tree	TRW	1565	2005	1	^131^	115	yes
Asi_102	GANGCD	30.98	78.93	tree	TRW	1567	1999	1	^132^	73	yes
Asi_103	RANGCD	33.08	76.43	tree	TRW	1388	2002	1	^132^	73	yes
Asi_104	INDI015	31.2	77.23	tree	TRW	1590	1990	1	^132^	116	yes
Asi_105	INDI025	10.18	76.87	tree	TRW	1481	2003	1	^132^	117	yes
Asi_106	KERALA	10	76.67	tree	TRW	1481	2003	1	^132^	73	yes
Asi_107	INDI024	31.37	78.17	tree	TRW	1538	2004	1	^132^	118	yes
Asi_108	JAPA008	43.77	142.55	tree	TRW	1532	1997	1	^132^	119	yes
Asi_109	JAPA008	43.77	142.55	tree	TRW	1557	1997	1	^132^	119	yes
Asi_110	RUSS219	43.88	145.6	tree	TRW	1585	2000	1	^131^	120	yes
Asi_111	JAPA020	30.33	130.5	tree	TRW	1	1999	1	^132^	121	yes
Asi_112	JAPA013	44.35	142.18	tree	TRW	1575	1999	1	^134^	122	yes
Asi_113	JAPA015	43.22	145.47	tree	TRW	1511	1998	1	^132^	123	yes
Asi_114	JAPA016	35.73	138.22	tree	TRW	1540	2001	1	^132^	124	yes
Asi_115	JAPA014	43.5	143.2	tree	TRW	1487	1997	1	^132^	125	yes
Asi_116	JAPA012	44.95	142.12	tree	TRW	1500	1991	1	^134^	126	yes
Asi_117	JAPA018	30.37	130.53	tree	TRW	1080	2005	1	^132^	127	yes
Asi_118	JAPA017	30.37	130.53	tree	TRW	1080	2002	1	^132^	128	yes
Asi_119	JAPA018	30.33	130.45	tree	TRW	1141	2005	1	^132^	127	yes
Asi_120	JAPA019	33.73	133.12	tree	TRW	1600	2001	1	^132^	129	yes
Asi_121	KYRG002	40.17	72.58	tree	TRW	1346	1995	1	^135^	130	yes
Asi_122	KYRG003	40.17	72.58	tree	TRW	1591	1995	1	^135^	131	yes
Asi_123	KYRG004	40.17	72.58	tree	TRW	1378	1995	1	^135^	132	yes
Asi_124	KYRG005	40.17	72.58	tree	TRW	1316	1995	1	^135^	133	yes
Asi_125	KYRG007	40.17	72.58	tree	TRW	1157	1995	1	^135^	134	yes
Asi_126	KYRG008	40.2	72.58	tree	TRW	1420	1995	1	^132^	135	yes
Asi_127	KYRG009	39.92	71.47	tree	TRW	1019	1995	1	^132^	136	yes
Asi_128	KYRG010	40.17	72.62	tree	TRW	1427	1995	1	^132^	137	yes
Asi_129	KYRG011	39.83	71.5	tree	TRW	694	1995	1	^132^	138	yes
Asi_130	SHIYAT	39.83	71.5	tree	TRW	870	1995	1	^132^	73	yes
Asi_131	ESPERG	40.17	72.58	tree	TRW	1340	1995	1	^132^	73	yes
Asi_132	SHIESP	39.83	71.5	tree	TRW	1340	1995	1	^132^	73	yes
Asi_133	RUSS236	55	160.5	tree	TRW	1580	2001	1	^136^	139	yes
Asi_134	KAZ001	43.35	77.35	tree	TRW	1570	2001	1	^137^	140	yes
Asi_135	KYRG012	42.2	79.05	tree	TRW	1450	2005	1	^132^	141	yes
Asi_136	KYRG013	42.15	79.47	tree	TRW	1301	2005	1	^132^	142	yes
Asi_137	KYRG014	42.42	78.97	tree	TRW	1551	2005	1	^132^	143	yes
Asi_138	KYRG015	42.15	79.45	tree	TRW	1528	2005	1	^132^	144	yes
Asi_139	MONG016	48.6	88.37	tree	TRW	1469	2004	1	^131^	145	yes
Asi_140	MONG027	46.32	101.32	tree	TRW	1599	2001	1	^131^	120	yes
Asi_141	MONG017	49.97	91	tree	TRW	1350	2005	1	^131^	120	yes
Asi_142	MONG018	49.97	90.98	tree	TRW	1519	2005	1	^131^	120	yes
Asi_143	MONG019	47.1	90.97	tree	TRW	1375	2004	1	^131^	120	yes
Asi_144	MONG020	48.27	88.87	tree	TRW	1537	2005	1	^131^	120	yes
Asi_145	MONG021	48.35	107.47	tree	TRW	996	2002	1	^131^	120	yes
Asi_146	MONG033	49.37	94.88	tree	TRW	1550	1997	1	^131^	120	yes
Asi_147	MONG009	49.92	91.57	tree	TRW	1326	1998	1	^132^	146	yes
Asi_148	MONG015	48.17	99.87	tree	TRW	1340	2000	1	^132^	146	yes
Asi_149	MONG024	48.5	88.5	tree	TRW	1215	2004	1	^131^	120	yes
Asi_150	MONG025	48.7	88.8	tree	TRW	1565	2004	1	^131^	120	yes
Asi_151	MONG026	46.82	100.12	tree	TRW	1411	2002	1	^131^	120	yes
Asi_152	MONG002	47.77	107	tree	TRW	1506	1994	1	^132^	146	yes
Asi_153	MONG028	48.83	111.68	tree	TRW	1576	2001	1	^131^	120	yes
Asi_154	MONG029	49.87	91.43	tree	TRW	1249	1998	1	^131^	120	yes
Asi_155	SODAPS	48.3	98.93	tree	TRW	46	1999	1	^132^	73	yes
Asi_156	MONG030	49.38	94.88	tree	TRW	1432	1998	1	^131^	120	yes
Asi_157	MONG010	47.27	100.03	tree	TRW	1363	1999	1	^132^	146	yes
Asi_158	MONG014	47.43	100.42	tree	TRW	1557	2002	1	^132^	146	yes
Asi_159	MONG007	49.7	91.55	tree	TRW	1570	1995	1	^132^	147	yes
Asi_160	MONG012	48.98	103.22	tree	TRW	1511	2002	1	^138^	146	yes
Asi_161	MONG031	48.25	97.4	tree	TRW	1516	1998	1	^131^	120	yes
Asi_162	MONG006	47.78	107.5	tree	TRW	1582	1996	1	^138^	146	yes
Asi_163	MONG011	48.13	100.27	tree	TRW	1513	2001	1	^138^	146	yes
Asi_164	MONG032	46.52	100.95	tree	TRW	1340	2002	1	^131^	120	yes
Asi_165	Hushre	46.78	101.95	tree	TRW	1503	2009	1	^132^	73	yes
Asi_166	MONG004	47.95	107.45	tree	TRW	1590	2002	1	^132^	146	yes
Asi_167	NEPA003	29.48	82.08	tree	TRW	1420	1997	1	^132^	148	yes
Asi_168	NEPA010	27.7	86.45	tree	TRW	1417	1998	1	^132^	149	yes
Asi_169	NEPA014	27.7	86.28	tree	TRW	1546	1998	1	^132^	150	yes
Asi_170	NEPA015	28.38	83.7	tree	TRW	1395	1997	1	^132^	151	yes
Asi_171	NEPA018	27.73	86.33	tree	TRW	1445	1998	1	^132^	152	yes
Asi_172	NEPA019	29.47	82.12	tree	TRW	1530	1997	1	^132^	153	yes
Asi_173	NEPA025	29.52	82.03	tree	TRW	1566	1997	1	^132^	154	yes
Asi_174	NEPA029	28.18	85.43	tree	TRW	1559	1994	1	^132^	155	yes
Asi_175	NEPA030	27.78	87.27	tree	TRW	856	1996	1	^132^	156	yes
Asi_176	NEPA032	27.67	87.2	tree	TRW	1546	1996	1	^132^	157	yes
Asi_177	NEPA036	27.73	87.2	tree	TRW	1509	1996	1	^132^	158	yes
Asi_178	BURGPW	28.77	83.73	tree	TRW	1303	1996	1	^132^	73	yes
Asi_179	NEPA021	27.5	88.02	tree	TRW	1525	1999	1	^132^	159	yes
Asi_180	NEPA027	27.5	87.98	tree	TRW	1561	1999	1	^132^	160	yes
Asi_181	NEPA042	27.83	88.02	tree	TRW	1500	1999	1	^131^	161	yes
Asi_182	ENEPAB	27.73	87.2	tree	TRW	1509	1999	1	^132^	73	yes
Asi_183	ESPPAK	35.17	75.5	tree	TRW	700	1993	1	^132^	73	yes
Asi_184	PAKI017	35.33	74.8	tree	TRW	1505	2005	1	^132^	162	yes
Asi_185	PAKI018	35.33	74.8	tree	TRW	1317	2005	1	^132^	163	yes
Asi_186	PAKI001	36.03	74.58	tree	TRW	1593	1993	1	^135^	164	yes
Asi_187	PAKI002	36.03	74.58	tree	TRW	1369	1993	1	^135^	165	yes
Asi_188	PAKI003	36.03	74.58	tree	TRW	1438	1993	1	^135^	166	yes
Asi_189	PAKI004	36.03	74.58	tree	TRW	1240	1993	1	^135^	167	yes
Asi_190	PAKI020	35.68	71.63	tree	TRW	1411	2006	1	^132^	168	yes
Asi_191	PAKI021	35.68	71.63	tree	TRW	1403	2006	1	^132^	169	yes
Asi_192	PAKI022	35.9	71.73	tree	TRW	1537	2006	1	^132^	170	yes
Asi_193	PAKI023	35.9	71.73	tree	TRW	1260	2006	1	^132^	171	yes
Asi_194	PAKI024	35.03	74.58	tree	TRW	1394	2005	1	^132^	172	yes
Asi_195	PAKI006	36.33	74.03	tree	TRW	1032	1993	1	^135^	173	yes
Asi_196	PAKI007	36.33	74.03	tree	TRW	1141	1993	1	^135^	174	yes
Asi_197	PAKI025	35.45	74.78	tree	TRW	1290	2007	1	^132^	175	yes
Asi_198	PAKI027	35.35	71.93	tree	TRW	1511	2006	1	^132^	176	yes
Asi_199	PAKI028	35.4	74.12	tree	TRW	1559	2007	1	^132^	177	yes
Asi_200	PAKI029	35.83	74.33	tree	TRW	1523	2008	1	^132^	178	yes
Asi_201	PAKI030	35.88	74.18	tree	TRW	1574	2008	1	^132^	179	yes
Asi_202	PAKI009	36.58	75.08	tree	TRW	476	1990	1	^135^	180	yes
Asi_203	PAKI010	36.58	75.08	tree	TRW	968	1990	1	^135^	181	yes
Asi_204	PAKI011	36.58	75.08	tree	TRW	554	1990	1	^135^	182	yes
Asi_205	PAKI012	36.58	75.08	tree	TRW	1069	1990	1	^135^	183	yes
Asi_206	PAKI031	35.5	74.08	tree	TRW	1296	2007	1	^132^	184	yes
Asi_207	PAKI033	35.5	74.75	tree	TRW	1362	2007	1	^132^	185	yes
Asi_208	PAKI035	36.15	74.18	tree	TRW	1497	2009	1	^132^	186	yes
Asi_209	PAKI036	36.15	74.18	tree	TRW	1387	2005	1	^132^	187	yes
Asi_210	PAKI014	35.17	75.5	tree	TRW	1412	1993	1	^135^	188	yes
Asi_211	PAKI015	35.17	75.5	tree	TRW	736	1993	1	^135^	189	yes
Asi_212	PAKI016	35.17	75.5	tree	TRW	388	1993	1	^135^	190	yes
Asi_213	PAKI039	35	70.78	tree	TRW	1569	2007	1	^132^	191	yes
Asi_214	PAKI040	35.35	71.8	tree	TRW	1472	2005	1	^139^	192	yes
Asi_215	HRPCSM	35.88	74.88	tree	TRW	1467	2009	1	^132^	73	yes
Asi_216	NOGSAK	51.83	143.13	tree	TRW	1570	2003	1	^132^	73	yes
Asi_217	CHIN042	31.95	98.87	tree	TRW	1519	1996	1	^132^	193	yes
Asi_218	CHIN047	31.12	97.03	tree	TRW	1406	1994	1	^132^	194	yes
Asi_219	CHIN049	31.23	96.48	tree	TRW	1547	1994	1	^132^	195	yes
Asi_220	CHIN043	29.3	91.97	tree	TRW	1217	1998	1	^132^	196	yes
Asi_221	CHIN046	31.12	97.03	tree	TRW	449	2004	1	^132^	197	yes
Asi_222	CHIN044	29.07	93.95	tree	TRW	1047	1993	1	^132^	198	yes
Asi_223	CHIN045	29.62	94.67	tree	TRW	1568	1993	1	^132^	199	yes
Asi_224	CHIN048	30.3	91.52	tree	TRW	1080	1998	1	^132^	200	yes
Asi_225	CENTIB	29.35	92	tree	TRW	1285	2008	1	^132^	73	yes
Asi_226	TH001	19.28	98.93	tree	TRW	1558	2005	1	^131^	201	yes
Asi_227	TW001	24.53	121.38	tree	TRW	907	2007	1	^131^	202	yes
Asi_228	MCCHFH	21.67	104.1	tree	TRW	1470	2004	1	^140^	139	yes
Asi_229	VIET001	12.22	108.73	tree	TRW	1030	2008	1	^141^	203	yes
Asi_230	Central China	29	113	documents	historic	1470	1990	10	^142^	1	yes
Asi_231	Sihailongwa lake	42.17	126.36	lake sediment	alkenone	392	2002	19	^143^	1	yes
Asi_232	Dasuopu	28.38	85.72	glacier ice	d18O	1450	1996	1	^144^	204	yes
Asi_233	East China	30	117.5	documents	historic	1470	1970	10	^145^	1	yes
Asi_234	East China region	34	120	documents	historic	1380	1990	10	^142^	1	yes
Asi_235	Fujian and Taiwan	24	121	documents	historic	1500	1960	10	^142^	1	yes
Asi_236	Guangdong	23.16	113.23	documents	historic	1470	1940	10	^146^	1	yes
Asi_237	Guangdong and Guangxi province	23.5	112.5	documents	historic	1470	1960	10	^147^	1	yes
Asi_238	Guliya	35.28	81.48	glacier ice	d18O	5	1985	10	^148^	205	yes
Asi_239	Hunan-Jiangsu	28	116.5	documents	historic	1470	1960	10	^147^	1	yes
Asi_240	Kunashir Island	43.955	145.725	hybrid	hybrid	1585	2000	1	^149^	1	yes
Asi_241	Lower reaches of the Yangtze River	32.1	118.8	documents	historic	1470	1960	10	^147^	1	yes
Asi_242	Middle reaches of the Yangtze River	30.5	114.5	documents	historic	1470	1960	10	^147^	1	yes
Asi_243	Puruogangri	33.9167	89.0833	glacier ice	d18O	5	1995	10	^150^	206	yes
Asi_244	Sourth and Middle Urals	55	59.5	borehole	borehole	800	1950	50	^151^	1	no
Asi_245	South China	23	114	documents	historic	1500	1960	10	^142^	1	yes
Asi_246	Zhejiang and Fujian province	25	118	documents	historic	1470	1960	10	^147^	1	yes
Aus_001	Mt. Read	−41.83	145.53	tree	TRW	−494	2001	1	^152^	207	yes
Aus_002	Oroko	−43.23	170.28	tree	TRW	900	1999	1	^153^	208	yes
Aus_004	CTP East Tasmania	−41.31	147.75	tree	TRW	1430	1994	1	^154^	209	yes
Aus_005	Pink Pine NZ	−43	171	tree	TRW	1457	1999	1	^155^	210	yes
Aus_007	Buckleys Chance Tasmania (Site ID: BCH)	−42.27	145.87	tree	TRW	1463	1991	1	^156^	211	yes
Aus_009	CTP West Tasmania	−41.67	145.65	tree	TRW	1547	1998	1	^154^	212	yes
Aus_029	Duckhole Lake	−43.3646	146.8749	lake sediment	reflectance	1140	2001	2	^157^	213	yes
Aus_030	Stewart Island	−47	167.8	tree	TRW	1758	1993	1	^158^	214	yes
Aus_031	Takapari Cedar	−40.07	175.98	tree	TRW	1530	1992	1	^159^	215	yes
Eur_003	Northern Scandinavia	68	25	tree	MXD	−138	2006	1	^160^	216	yes
Eur_004	Tatra Mountains	49	20	tree	TRW	1040	2011	1	^161^	217	yes
Eur_005	Eastern Carpathian Mountains (Romania)	47	25.3	tree	TRW	1163	2005	1	^162^	218	yes
Eur_006	European Alps	47	10.7	tree	TRW	−500	2003	1	^163^	219	yes
Eur_007	Lötschental	46.4	7.8	tree	MXD	755	2004	1	^164^	220	yes
Eur_008	Maritime French Alps	44	7.5	tree	TRW	969	2007	1	^165^	221	yes
Eur_009	Spanish Pyrenees	42.5	1	tree	TRW	1260	2005	1	^166^	222	yes
Eur_011	Central Europe	49	13	documents	TRW	1500	2007	1	^167^	223	yes
Eur_012	Central and Eastern Pyrenees (NE Spain)	42.5	0.75	lake sediment	chrysophyte	578	1994	25	^168^	1	yes
Eur_013	Finnish Lakelands	62	28.325	tree	MXD	760	2000	1	^169^	224	yes
Eur_014	Lake Silvaplana	46.5	9.8	lake sediment	chironomid	1032	1975	1	^170^	225	yes
Eur_015	Lake Silvaplana	46.5	9.8	lake sediment	reflectance	1175	1949	1	^171^	226	yes
Eur_016	Seebergsee	46.15	7.5	lake sediment	midge	1083	2001	1	^172^	227	yes
Eur_017	Northern Spain	42.9	−3.5	speleothem	d18O	−1949	2000	3	^173^	228	yes
Eur_018	Spannagel Cave,	47.1	11.6	speleothem	d18O	−90	1935	3	^174^	229	yes
Eur_019	Stockholm	59.32	18.06	documents	historic	1502	1892	1	^175^	230	yes
Eur_020	Tallinn	59.4	24.75	documents	historic	1500	2000	1	^176^	1	yes
NAm_001	San Franciso Peaks Update	35.3	−111.4	tree	TRW	1	2002	1	^177^	231	yes
NAm_002	Kobuk/Noatak	67.1	−159.6	tree	TRW	978	1992	1	no publication	232	yes
NAm_003	Prince William Sound	60.5	−148.3	tree	TRW	873	1991	1	^178^	233	yes
NAm_007	Flower Lake	36.5	−118.2	tree	TRW	898	1987	1	no publication	234	yes
NAm_008	Timber Gap Upper	36.3	−118.4	tree	TRW	699	1987	1	no publication	235	yes
NAm_009	Cirque Peak	36.3	−118.2	tree	TRW	917	1987	1	no publication	236	yes
NAm_011	Sheep Mountain	37.2	−118.1	tree	TRW	−1	1990	1	no publication	237	yes
NAm_013	Spillway Lake Yosemite National Park	37.8	−119.2	tree	TRW	800	1996	1	no publication	238	yes
NAm_018	Boreal Plateau	36.3	−118.3	tree	TRW	831	1992	1	^179^	239	yes
NAm_019	Upper Wright Lakes	36.4	−118.2	tree	TRW	−215	1992	1	^179^	240	yes
NAm_026	Athabasca, historisch	51.4	−117.3	tree	MXD	1072	1991	1	^180^	241	yes
NAm_029	Bennington	52.7	−118.3	tree	TRW	1104	1996	1	^181^	242	yes
NAm_030	French Glacier	50.8	−115.3	tree	TRW	1069	1993	1	^182^	243	yes
NAm_032	Landslide	60.2	−138.5	tree	TRW	913	2001	1	^183^	244	yes
NAm_041	Fool Creek	39.9	−105.9	tree	MXD	1296	1993	1	no publication	245	yes
NAm_044	Yellow Mountain Ridge	45.3	−111.3	tree	TRW	470	1998	1	no publication	246	yes
NAm_045	Flint Creek Range	46.3	−113.2	tree	TRW	999	1998	1	no publication	247	yes
NAm_046	Pintlers	46	−113.4	tree	TRW	1200	2005	1	^184^	248	yes
NAm_049	Pearl Peak	40.2	−115.5	tree	TRW	320	1985	1	no publication	249	yes
NAm_050	Mount Washington	38.5	−114.2	tree	TRW	825	1983	1	no publication	250	yes
NAm_059	Granite Mountain	47.3	−121.3	tree	TRW	1259	1980	1	no publication	251	yes
NAm_060	Scatter Lake	48.3	−120.3	tree	TRW	1316	1980	1	no publication	252	yes
NAm_064	Sylvan Pass bei Cody	44.4	−110.1	tree	MXD	1388	1983	1	^180^	253	yes
NAm_065	Basin Pond	44.5	−70.1	lake sediment	pollen	362	1960	31	^185^	254	yes
NAm_066	Carlton Ridge	46.7	−114.2	tree	TRW	1150	1998	1	^186^	1	yes
NAm_067	Clear Pond	33.8	−79	lake sediment	pollen	52	1960	39	^185^	254	yes
NAm_068	Conroy Lake	46.3	−67.9	lake sediment	pollen	10	1967	33	^185^	254	yes
NAm_069	Dark Lake	45.3	−91.5	lake sediment	pollen	1044	1950	18	^185^	254	yes
NAm_070	Fremont Glacier	43	−109.6	tree	TRW	1227	2001	1	no publication	1	yes
NAm_071	Great Basin 4500yr Composite Chronology	37	−116.5	tree	TRW	1	2009	1	^187^	1	yes
NAm_072	Green Lake	50.2	−122.9	lake sediment	varve thickness	1388	1999	1	^188^	1	yes
NAm_073	Hell’s Kitchen Lake	46.2	−89.7	lake sediment	pollen	−53	1960	22	^185^	254	yes
NAm_074	Lake of the Clouds	48	−91	lake sediment	pollen	1030	1970	10	^185^	254	yes
NAm_075	Lac Noir	45.8	−75.1	lake sediment	pollen	1007	2007	10	^189^	255	yes
NAm_076	Lake Mina	45.89	−95.478	lake sediment	pollen	1120	1900	4	^190^	256	yes
NAm_077	Lake Mina	45.89	−95.478	lake sediment	pollen	1120	1900	4	^190^	256	yes
NAm_078	LittlePine Lake	45.3	−91.5	lake sediment	pollen	1237	1950	18	^185^	254	yes
NAm_079	Oregon Caves National Monument	42.083	−123.416	speleothem	d18O	−6015	1716	2	^191^	257	yes
NAm_080	Ruby Lake	45.3	−91.5	lake sediment	pollen	1121	1965	17	^185^	254	yes
NAm_081	Siberian Outpost View	36.5	−118.3	tree	TRW	494	2001	1	^192^	1	yes
NAm_082	L1_CANA458, L12_CANA459, L16_CANA460, L18_CANA461, L20_CANA462, L22_CANA463	54.21	−71.35	tree	TRW	910	2011	1	^193^	258	yes
NAm_083	Herring Alpine	60.4	−147.8	tree	TRW	1422	1972	1	no publication	259	yes
NAm_084	Denali National Park	63.7	−149.6	tree	MXD	1551	1983	1	^180^	260	yes
NAm_085	Eureka Summit	61.8	−147.3	tree	TRW	1654	1983	1	^180^	261	yes
NAm_086	Eureka Summit	61.8	−147.3	tree	MXD	1654	1983	1	^180^	261	yes
NAm_087	Settlement Point Afognak Island	58.1	−152.7	tree	TRW	1690	1996	1	no publication	262	yes
NAm_088	Miners Well	60	−141.7	tree	TRW	1428	1995	1	no publication	263	yes
NAm_089	Canyon Creek	63.3	−147.8	tree	TRW	1642	1997	1	no publication	264	yes
NAm_090	Almond Butter Lower	65.2	−162.2	tree	TRW	1607	2002	1	^194^	265	yes
NAm_091	Almond Butter Upper	65.2	−162.2	tree	TRW	1406	2002	1	^194^	266	yes
NAm_092	Burnt Over	65.2	−162.3	tree	TRW	1621	2002	1	^194^	267	yes
NAm_093	Windy Ridge Alaska	65.2	−162.2	tree	TRW	1556	2002	1	^194^	268	yes
NAm_094	Nabesna Mine	62.4	−143.1	tree	TRW	1471	1997	1	^195^	269	yes
NAm_095	Nabesna Mine	62.4	−143.1	tree	MXD	1540	1997	1	^195^	269	yes
NAm_096	Big Bend Lake	61.3	−142.7	tree	TRW	1557	1997	1	^195^	270	yes
NAm_097	Deer Mountain	55.3	−131.6	tree	TRW	1550	1999	1	no publication	271	yes
NAm_098	McGinnis Trail	58.4	−134.6	tree	TRW	1380	1999	1	^196^	272	yes
NAm_099	Southern Alaska composite	56	−132	tree	TRW	1343	2000	1	^87^	273	yes
NAm_100	Seward Composite	65.2	−162.3	tree	TRW	1389	2001	1	^87^	274	yes
NAm_101	Seward Composite	65.2	−162.3	tree	MXD	1389	2001	1	^87^	274	yes
NAm_102	Wrangells Composite	62	−142	tree	MXD	1540	1998	1	^87^	275	yes
NAm_103	Northern Alaska Composite	67	−152	tree	MXD	1524	1990	1	^87^	276	yes
NAm_104	Firth River 1236	68.7	−141.6	tree	MXD	1073	2002	1	^197^	277	yes
NAm_105	Starrigawan Old Sitka	57.1	−135.4	tree	TRW	1605	1985	1	no publication	278	yes
NAm_106	Snow Bowl San Francisco Peak	35.4	−110.2	tree	MXD	1453	1983	1	^130^	279	yes
NAm_107	Mt. Lemon	32.5	−110.8	tree	MXD	1568	1983	1	^130^	280	yes
NAm_108	Yosemite Park E Eingang	37.8	−119.3	tree	MXD	1513	1983	1	^180^	281	yes
NAm_109	Dana Plateau Inyo National Forest	37.9	−119.2	tree	TRW	1430	1996	1	no publication	282	yes
NAm_110	Fish Creek Trail (San Gorgonio)	34.1	−116.8	tree	TRW	1534	1995	1	no publication	283	yes
NAm_111	Fort Chimo (Merged)	58.4	−68.4	tree	TRW	1641	1974	1	^198^	284	yes
NAm_112	Spruce Creek	68.6	−138.6	tree	TRW	1570	1977	1	^198^	285	yes
NAm_113	Pethai Peninsula	62.7	−111	tree	MXD	1610	1988	1	^180^	286	yes
NAm_114	Lac Romanel (Feucht)	56.2	−67.7	tree	TRW	1659	1988	1	^180^	287	yes
NAm_115	Lac Romanel (Feucht)	56.2	−67.7	tree	MXD	1659	1988	1	^180^	287	yes
NAm_116	Don Jek River Bridge	61.7	−139.7	tree	MXD	1585	1983	1	^180^	288	yes
NAm_117	Smithers Ski Area	54.9	−127.3	tree	TRW	1680	1983	1	^180^	289	yes
NAm_118	Smithers Ski Area	54.9	−127.3	tree	MXD	1680	1983	1	^180^	289	yes
NAm_119	Bell Mountain	53.3	−120.7	tree	MXD	1649	1983	1	^180^	290	yes
NAm_120	Sunwapta Pass	52.3	−117	tree	TRW	1608	1983	1	^180^	291	yes
NAm_121	Sunwapta Pass	52.3	−117	tree	MXD	1608	1983	1	^180^	291	yes
NAm_122	Peyto Lake	51.8	−116.2	tree	MXD	1634	1983	1	^180^	292	yes
NAm_123	Vancouver Cyprus Provincial Park	49.4	−123.1	tree	MXD	1413	1983	1	^180^	293	yes
NAm_124	Arrowsmith Mountain	49.2	−125.2	tree	MXD	1629	1983	1	^180^	294	yes
NAm_125	Big White	49.9	−118.9	tree	TRW	1669	1998	1	no publication	295	yes
NAm_126	Coppermine River	67.2	−115.9	tree	TRW	1428	1977	1	^1^	296	yes
NAm_127	Hornby Cabin	64	−103.9	tree	TRW	1491	1984	1	^1^	297	yes
NAm_128	Twisted Tree Heartrot Hill	65	−138.3	tree	TRW	1550	1975	1	^1^	298	yes
NAm_129	Bonif historisc	55.3	−77.8	tree	TRW	1352	1989	1	^180^	299	yes
NAm_130	Bonif historisc	55.3	−77.8	tree	MXD	1352	1989	1	^180^	299	yes
NAm_131	Athabasca Glacier	52.3	−117.3	tree	MXD	1665	1994	1	^180^	300	yes
NAm_132	Medusa Bay	56.9	−61.5	tree	TRW	1634	1997	1	^199^	301	yes
NAm_133	Meadow Mountain	50.2	−117.1	tree	TRW	1669	1997	1	^200^	302	yes
NAm_134	Meadow Mountain	50.2	−117.1	tree	MXD	1685	1997	1	^200^	302	yes
NAm_135	Ymir	49.4	−117.2	tree	TRW	1693	1997	1	^200^	303	yes
NAm_136	Park Mountain	50.6	−118.6	tree	TRW	1477	1997	1	^200^	304	yes
NAm_137	Park Mountain	50.6	−118.6	tree	MXD	1528	1997	1	^200^	304	yes
NAm_138	Big White 2	49.7	−118.9	tree	TRW	1580	1997	1	^200^	305	yes
NAm_139	Big White 2	49.7	−118.9	tree	MXD	1512	1997	1	^200^	305	yes
NAm_140	Cornwall Hills	50.7	−121.5	tree	TRW	1554	1997	1	^200^	306	yes
NAm_141	Cornwall Hills	50.7	−121.5	tree	MXD	1689	1997	1	^200^	306	yes
NAm_142	Ittyhauk Bay	56	−61	tree	TRW	1689	1998	1	no publication	307	yes
NAm_143	Eagle Fecal/Ptarmigan Merge	69.5	−127.8	tree	MXD	1550	1993	1	no publication	308	yes
NAm_144	Manitoba composite	58	−94	tree	TRW	1650	1982	1	^87^	309	yes
NAm_145	Blanchard River	59.9	−136.8	tree	TRW	1670	2003	1	no publication	310	yes
NAm_146	Nadahini	59.8	−136.6	tree	TRW	1572	2003	1	no publication	311	yes
NAm_147	Rock Glacier Yukon	61.4	−128.4	tree	TRW	1697	2002	1	no publication	312	yes
NAm_148	Snake Creek	59.6	−133.4	tree	TRW	1645	1999	1	no publication	313	yes
NAm_149	Sugarloaf	60.1	−134.4	tree	TRW	1700	2003	1	no publication	314	yes
NAm_150	Sunshine Meadows	51.1	−115.8	tree	MXD	1440	1987	1	^182^	315	yes
NAm_151	Athabasca Glacier 2	52.2	−117.2	tree	TRW	920	1987	1	^200^	316	yes
NAm_152	Athabasca Glacier 2	52.2	−117.2	tree	MXD	1665	1994	1	^200^	316	yes
NAm_153	Bennington	52.7	−118.3	tree	TRW	1563	1996	1	^201^	317	yes
NAm_154	Bow Summit/Peyto Lake	51.7	−116.5	tree	TRW	1575	2000	1	^200^	318	yes
NAm_155	Cardinal Divide	52.9	−117.3	tree	TRW	1528	1990	1	^201^	319	yes
NAm_156	Geraldine Lakes	52.6	−117.9	tree	TRW	1513	1990	1	^201^	320	yes
NAm_157	Highwood Pass	50.6	−115	tree	TRW	1613	1987	1	^201^	321	yes
NAm_158	Hilda	52.2	−117.2	tree	TRW	1428	2000	1	^202^	322	yes
NAm_159	Nakiska	50.9	−115.2	tree	TRW	1619	1987	1	^201^	323	yes
NAm_160	Pyramid Mountain	53	−118.2	tree	TRW	1625	1990	1	^201^	324	yes
NAm_161	Signal Mountain	52.9	−118	tree	TRW	1489	1990	1	^201^	325	yes
NAm_162	Small River	53.2	−119.5	tree	TRW	1569	1989	1	^201^	326	yes
NAm_163	Surprise Valley	52.8	−117.7	tree	TRW	1600	1990	1	^201^	327	yes
NAm_164	Arapahoe	40.1	−105.6	tree	MXD	1610	1982	1	no publication	328	yes
NAm_165	Red Mountain Pass Silverton	37.9	−107.7	tree	MXD	1626	1983	1	no publication	329	yes
NAm_166	Pike Peaks	39.3	−105	tree	TRW	1530	1983	1	^180^	330	yes
NAm_167	Pike Peaks	39.3	−105	tree	MXD	1530	1983	1	^180^	330	yes
NAm_168	Cottonwood Pass	38.7	−107.6	tree	TRW	1565	1982	1	^180^	331	yes
NAm_169	Cottonwood Pass	38.7	−107.6	tree	MXD	1565	1982	1	^180^	331	yes
NAm_170	Fool Creek	39.9	−105.9	tree	TRW	1224	1992	1	no publication	245	yes
NAm_171	Cameron Pass	40.6	−105.8	tree	TRW	1552	2003	1	^203^	332	yes
NAm_172	Galena Pass Sawtooth National Forest	43.9	−114.7	tree	MXD	1530	1983	1	^180^	333	yes
NAm_173	Sleeping Deer Road	44.6	−114.5	tree	TRW	1305	1997	1	no publication	334	yes
NAm_174	Sleeping Deer Road	44.6	−114.5	tree	MXD	1488	1997	1	no publication	334	yes
NAm_175	Elephant Mountain	44.8	−70.8	tree	MXD	1667	1977	1	no publication	335	yes
NAm_176	La Tasajera (San Pedro Martir)	31	−115.5	tree	TRW	1536	1995	1	no publication	336	yes
NAm_177	Cienega de Nuestra Senora de Guadalupe	25.1	−106.3	tree	TRW	1675	1993	1	no publication	337	yes
NAm_178	Highland Fire Outlook	45.8	−112.5	tree	TRW	1496	1983	1	^180^	338	yes
NAm_179	Highland Fire Outlook	45.8	−112.5	tree	MXD	1496	1983	1	^180^	338	yes
NAm_180	Baker Lake	45.9	−114.3	tree	TRW	12	1997	1	no publication	339	yes
NAm_181	Baker Lake	45.9	−114.3	tree	MXD	1434	1997	1	no publication	339	yes
NAm_182	Pintlers Two	46	−113.4	tree	TRW	1248	2006	1	^184^	340	yes
NAm_183	Rowe Lakes	49	−114	tree	TRW	1252	1990	1	^184^	341	yes
NAm_184	Crater Lake NE	43	−122.2	tree	MXD	1564	1983	1	^130^	342	yes
NAm_185	Barlow Pass am Mt.Hood	45.3	−121.7	tree	MXD	1504	1983	1	^180^	343	yes
NAm_186	Tamarack Bowl	49.3	−114.4	tree	TRW	1616	2012	1	no publication	344	yes
NAm_187	Ceader Breaks	37.6	−113.9	tree	MXD	1581	1983	1	^180^	345	yes
NAm_188	Vicary Mine	49.8	−114.5	tree	TRW	1488	2010	1	no publication	346	yes
NAm_189	Harts Pass N1	48.7	−120.7	tree	TRW	1685	1991	1	no publication	347	yes
NAm_190	Harts Pass N1	48.7	−120.7	tree	TRW	1585	1991	1	no publication	348	yes
NAm_191	Harts Pass N2	48.7	−120.7	tree	TRW	1529	1990	1	no publication	349	yes
NAm_192	Mt. St. Helens	46.2	−122.2	tree	MXD	1609	1983	1	^180^	350	yes
NAm_193	Sherman Creek Pass	48.7	−118.3	tree	TRW	1605	1983	1	^180^	351	yes
NAm_194	Sherman Creek Pass	48.7	−118.3	tree	MXD	1605	1983	1	^180^	351	yes
NAm_195	Hoh Lake High	47.9	−123.8	tree	TRW	1570	1992	1	no publication	352	yes
NAm_196	Mount Adams Low	46.2	−121.5	tree	TRW	1610	1992	1	no publication	353	yes
NAm_197	Powder River Pass	44.2	−107.1	tree	MXD	1496	1983	1	^180^	354	yes
NAm_198	Medicine Bow Peak	41.3	−107.7	tree	TRW	1401	1983	1	^180^	355	yes
NAm_199	Medicine Bow Peak	41.3	−107.7	tree	MXD	1401	1983	1	^180^	355	yes
NAm_200	Granite Pass Hunt Mountain	44.8	−107.9	tree	MXD	1508	1983	1	^180^	356	yes
NAm_201	Togwatee Pass	43.7	−110.1	tree	TRW	1672	1983	1	^180^	357	yes
NAm_202	Togwatee Pass	43.7	−110.1	tree	MXD	1672	1983	1	^180^	357	yes
NAm_203	Sheep Trail	41.4	−106.2	tree	TRW	1097	1999	1	no publication	358	yes
NAm_204	Sheep Trail	41.4	−106.2	tree	MXD	1483	2000	1	no publication	358	yes
Ocn_001	MD992275	66.55	−17.7	marine sediment	diatom	157	1952	17	^204^	359	yes
Ocn_002	Storegga Slide	63.76	5.26	marine sediment	foram d18O	−957	1999	7	^205^	1	yes
Ocn_003	Vøring Plateau	66.97	7.64	marine sediment	diatom	−4076	1995	12	^206^	360	yes
Ocn_004	Makassar Strait	−5.2012	117.4867	marine sediment	foram Mg/Ca	−40	1815	8	^207^	361	yes
Ocn_005	Alboran Sea	36.2053	−4.3133	marine sediment	alkenone	564	1999	32	^208^	362	yes
Ocn_006	Arabian Sea	24.83	65.92	marine sediment	alkenone	−216	1950	18	^209^	363	yes
Ocn_007	CH07-98-MC-22, Carolina Slope, western North Atlantic	32.784	−76.276	marine sediment	foram Mg/Ca	250	1850	100	^210^	364	yes
Ocn_008	Cape Ghir, NW Africa	30.85	−10.2685	marine sediment	alkenone	754	1950	37	^211^	365	yes
Ocn_009	Cape Ghir, NW Africa	30.845	−10.0983	marine sediment	alkenone	−225	1998	12	^212^	366	yes
Ocn_011	Cariaco Basin	10.77	−64.77	marine sediment	foram Mg/Ca	1221	1990	1	^213^	367	yes
Ocn_013	Cariaco Basin/Southern Caribbean Sea	10.7	−64.94	marine sediment	foram Mg/Ca	−158	1399	145	^214^	368	yes
Ocn_014	Chilean margin, Southern Ocean (Pacific sector)	−41	−74.45	marine sediment	alkenone	4	1650	76	^215^	369	yes
Ocn_015	Dry Tortugas	24.59	−83.58	marine sediment	foram Mg/Ca	659	1949	22	^43^	370	yes
Ocn_016	Dry Tortugas	24.33	−83.26	marine sediment	foram Mg/Ca	995	1925	23	^43^	370	yes
Ocn_017	Eastern tropical North Atlantic	16.8402	−16.7327	marine sediment	foram Mg/Ca	−236	2004	9	^216^	371	yes
Ocn_018	Emerald Basin,OCE326 MC-29D	45.89	−62.8	marine sediment	alkenone	351	1950	32	^217^	372	yes
Ocn_019	Emerald Basin, Nova Scotia	43.53	−62.48	marine sediment	alkenone	400	1950	110	^218^	373	yes
Ocn_020	Feni Drift	55.5	−13.9	marine sediment	foram Mg/Ca	−368	1998	22	^219^	374	yes
Ocn_021	Fisk Basin, Gulf of Mexico	27.55	−93.93	marine sediment	foram Mg/Ca	1217	1950	19	^220^	375	yes
Ocn_022	Garrison Basin, Gulf of Mexico	26.68	−93.93	marine sediment	foram Mg/Ca	1371	1950	24	^220^	375	yes
Ocn_023	Great Bahama Bank	24.58	−79.26	marine sediment	foram Mg/Ca	1065	1950	26	^43^	374	yes
Ocn_024	Great Bahama bank, 125MC	24.765	−79.29	marine sediment	foram Mg/Ca	590	1950	34	^43^	370	yes
Ocn_025	Gulf of Guinea	2.5	9.38	marine sediment	foram Mg/Ca	40	1590	30	^221^	376	yes
Ocn_027	Jacaf Fjord	−44.33	−72.97	marine sediment	alkenone	212	1784	24	^222^	377	yes
Ocn_028	KNR140‐2‐59GGC, Carolina Slope, western North Atlantic	32.977	−76.316	marine sediment	foram Mg/Ca	450	1850	50	^210^	364	yes
Ocn_029	Kuroshio Current	36.03	141.78	marine sediment	alkenone	−233	1947	26	^223^	378	yes
Ocn_030	Laurentian Fan, western subpolar North Atlantic	43.48	−54.87	marine sediment	alkenone	−230	1850	130	^224^	373	yes
Ocn_032	MD95-2011	66.97	7.63	marine sediment	alkenone	−290	1390	30	^225^	379	yes
Ocn_033	Makassar Strait	−7.4	115.2	marine sediment	foram Mg/Ca	175	1781	35	^226^	380	yes
Ocn_034	Makassar Strait	1.4033	119.078	marine sediment	foram Mg/Ca	160	1890	14	^207^	361	yes
Ocn_035	Makassar Strait	−3.53	119.2	marine sediment	foram Mg/Ca	−335	1982	7	^227^	381	yes
Ocn_036	Minorca contourite	40.5	4.03	marine sediment	alkenone	428	1988	15	^228^	382	yes
Ocn_037	Northwest Pacific Ocean	46.32	152.53	marine sediment	alkenone	−70	1370	50	^229^	383	yes
Ocn_039	MD992275	66.55	−17.7	marine sediment	alkenone	1	2001	4	^230^	384	yes
Ocn_040	ODP984	61.43	−24.085	marine sediment	foram Mg/Ca	152	1396	58	^231^	385	yes
Ocn_041	Okinawa Trough (ODP Hole 195−1202B)	24.8	122.5	marine sediment	TEX86	−227	1770	25	^232^	386	yes
Ocn_043	Pigmy Basin, Gulf of Mexico box core PBBC-1	27.2	−91.42	marine sediment	foram Mg/Ca	593	1901	12	^12^	387	yes
Ocn_045	RAPiD-12-1K, South Iceland Rise	62.08	−17.82	marine sediment	foram Mg/Ca	154	1252	73	^233^	388	yes
Ocn_046	SW coast of India	10.98	74.9993	marine sediment	foram Mg/Ca	303	1835	88	^234^	389	yes
Ocn_047	Santa Barbara Basin (California)	34.23	−120.02	marine sediment	alkenone	1298	1941	1	^235^	390	yes
Ocn_048	South Atlantic, West Africa	−29.14	16.72	marine sediment	alkenone	705	1938	20	^236^	391	yes
Ocn_049	South China Sea	8.73	109.869	marine sediment	alkenone	−182	993	26	^237^	392	yes
Ocn_050	South Iceland	57.45	−27.91	marine sediment	alkenone	−5	1724	7	^230^	384	yes
Ocn_051	Southern Chile Margin	−44.149	−75.16	marine sediment	alkenone	−168	1901	32	^238^	393	yes
Ocn_052	subtropical eastern North Atlantic	20.75	−18.5833	marine sediment	planktonic foraminifera	−140	1470	47	^239^	394	yes
Ocn_053	Tagus mud patch, southern portuguese margin, North Atlantic	38.556	−9.3498	marine sediment	alkenone	74	2001	3	^240^	395	no
Ocn_054	WEq Pacific	−5	133.44	marine sediment	foram Mg/Ca	685	1416	43	^241^	396	yes
Ocn_055	West Spitzberg, Fram Strait	78.96	5.885	marine sediment	dynocist MAT	−289	1943	43	^242^	397	yes
Ocn_056	Western Antarctic Peninsula	−64.87	−64.2	marine sediment	TEX86	−78	1870	50	^243^	398	yes
Ocn_058	Western Svalbard	78.91	6.77	marine sediment	planktonic foraminifera	110	2007	50	^244^	399	yes
Ocn_059	Alboran Sea	35.986	−4.7496	marine sediment	alkenone	980	2005	33	^208^	362	yes
Ocn_060	Biscayne Bay	25.38	−80.17	coral	d18O	1751	1986	1	^245^	400	yes
Ocn_061	Mayotte	−12.65	45.1	coral	Sr/Ca	1882	1994	<1	^246^	401	no
Ocn_062	Mayotte	−12.65	45.1	coral	d18O	1882	1994	<1	^246^	401	no
Ocn_063	Cape Hatteras	34.973	−75.201	marine sediment	foram Mg/Ca	−75	1294	10	^247^	402	yes
Ocn_064	Philippines	6.3	125.82	marine sediment	foram Mg/Ca	647	1909	17	^241^	403	yes
Ocn_065	Gingerbreads Bahamas	25.84	−78.62	coral	calcification	1552	1991	1	^248^	404	yes
Ocn_066	Bermuda south shore	30.6486	−64.9888	coral	d18O	1782	1998	1	^249^	405	yes
Ocn_067	Bermuda south shore	30.6486	−64.9888	coral	Sr/Ca	1781	1998	1	^249^	405	yes
Ocn_068	North East Breakers, Bermuda	32.467	−64.7	coral	d18O	1825	1983	<1	^250^	406	yes
Ocn_069	North East Breakers, Bermuda	32.467	−64.7	coral	Sr/Ca	1867	1983	<1	^250^	406	no
Ocn_070	Dry Tortugas	24.6	−82.3	coral	Sr/Ca	1734	2009	<1	^251^	407	yes
Ocn_071	Guadeloupe	16.2	−61.49	coral	d18O	1896	1999	<1	^252^	408	no
Ocn_072	Guadeloupe	16.2	−61.49	coral	Sr/Ca	1896	1999	<1	^252^	408	no
Ocn_073	Punta Maroma, Mexico	20.83	−86.74	coral	calcification	1773	2009	1	^253^	409	yes
Ocn_074	Lombok	−8.2473	115.5757	coral	d18O	1782	1990	<1	^254^	410	yes
Ocn_075	Ifaty	−23.15	43.58	coral	d18O	1660	1996	<1	^255^	411	yes
Ocn_076	Malindi	−3.2	40.1	coral	d18O	1887	2003	<1	^256^	412	no
Ocn_077	Mafia	−8.0167	39.5	coral	d18O	1622	1998	<1	^257^	413	yes
Ocn_078	Malindi	−3.2	40.1	coral	d18O	1801	1994	1	^258^	414	yes
Ocn_079	Mentawai Islands	−0.13	98.52	coral	d18O	1858	1998	<1	^259^	415	no
Ocn_080	Red Sea	27.85	34.32	coral	d18O	1751	1996	<1	^260^	416	yes
Ocn_081	Reunion	−21.0333	55.25	coral	d18O	1832	1996	<1	^261^	417	yes
Ocn_082	Sinai Peninsula, Red Sea	27.8483	34.31	coral	d18O	1897	1993	<1	^262^	418	no
Ocn_083	Mahe	−4.62	55	coral	d18O	1847	1995	<1	^263^	419	yes
Ocn_084	New Ireland, Papua	−2.5	150.5	coral	Sr/Ca	1823	1997	<1	^264^	420	yes
Ocn_086	Bunaken Island	−1.5	124.833	coral	d18O	1860	1991	<1	^254^	410	no
Ocn_087	Urvina Bay	−0.4	−91.23	coral	d18O	1607	1981	1	^265^	421	yes
Ocn_088	Double Reef	13.5982	144.8359	coral	d18O	1790	2000	<1	^266^	422	yes
Ocn_090	Laing Island, Papua New Guinea	−4.15	144.8833	coral	d18O	1885	1993	<1	^267^	423	no
Ocn_091	Savusavu Bay	−16.8167	179.2333	coral	d18O	1782	1997	1	^268^	424	yes
Ocn_093	Savusavu Bay	−16.8167	179.2333	coral	Sr/Ca	1782	1997	1	^268^	424	yes
Ocn_095	Savusavu Bay	−16.8167	179.2333	coral	d18O	1618	2002	1	^268^	424	yes
Ocn_096	Rarotonga	−21.2378	−159.8278	coral	Sr/Ca	1727	1997	<1	^268^	425	yes
Ocn_097	Madang Lagoon, Papua New Guinea	−5.2167	145.8167	coral	d18O	1881	1993	<1	^267^	426	no
Ocn_098	Maiana	1	173	coral	d18O	1840	1995	<1	^269^	427	yes
Ocn_099	Moorea	−17.5	−149.8333	coral	d18O	1853	1991	1	^270^	428	no
Ocn_101	Amedee Island	−22.475	166.4667	coral	Sr/Ca	1649	2000	<1	^271^	429	yes
Ocn_103	Palmyra	5.87	−162.13	coral	d18O	928	1998	<1	^272^	430	yes
Ocn_104	Secas Island, Panama	7.95	−82	coral	d18O	1708	1985	<1	^273^	431	yes
Ocn_106	Savusavu Bay, Fiji	−16.82	179.23	coral	d18O	1776	2001	1	^274^	432	yes
Ocn_107	Tarawa	1	172	coral	d18O	1894	1990	<1	^275^	433	no
Ocn_108	Vanuatu	−15	166.99	coral	d18O	1807	1979	1	^276^	434	yes
Ocn_109	Hafera Island	−19.9333	−174.7167	coral	Sr/Ca	1791	2003	1	^277^	435	yes
Ocn_110	Florida Bay	24.93	−80.75	coral	d18O	1824	1985	1	^278^	436	yes
Ocn_111	Turrumote Reef, Puerto Rico	17.93	−67	coral	d18O	1751	2004	1	^279^	437	yes
Ocn_112	Turrumote Reef, Puerto Rico	17.93	−67	coral	Sr/Ca	1751	2004	1	^279^	437	yes
Ocn_114	Houtman Abrolhos	−28.47	113.77	coral	d18O	1795	1994	<1	^41^	438	yes
Ocn_115	Aqaba, Jordan	29.42	34.97	coral	d18O	1788	1992	1	^280^	439	yes
Ocn_116	Aqaba, Jordan	29.42	34.97	coral	d18O	1886	1992	1	^280^	439	no
Ocn_118	Ningaloo	−21.905	113.965	coral	d18O	1879	1995	<1	^281^	440	no
Ocn_119	Clipperton Atoll	10.2773	−109.2131	coral	d18O	1894	1994	<1	^282^	441	no
Ocn_120	Miyanohama	27.1059	142.1941	coral	d18O	1872	1995	<1	^283^	442	no
Ocn_121	Miyanohama	27.1059	142.1941	coral	Sr/Ca	1872	1995	<1	^283^	442	no
Ocn_122	Abraham Reef	−22.1	153	coral	d18O	1638	1983	1	^284^	443	yes
Ocn_123	Vanuatu	−15.94	166.04	coral	d18O	1843	2008	<1	^285^	444	yes
Ocn_125	Rarotonga	−21.2378	−159.8278	coral	d18O	1727	1997	<1	^268^	425	yes
Ocn_127	Rarotonga	−21.2378	−159.8278	coral	d18O	1875	2000	<1	^268^	425	no
Ocn_128	Nauru	−0.53296	166.9283	coral	d18O	1898	1995	<1	^286^	445	no
Ocn_129	Palmyra	5.87	−162.13	coral	Sr/Ca	1886	1998	<1	^57^	446	no
Ocn_130	Rabaul	−4.1916	151.9772	coral	d18O	1867	1998	<1	^287^	447	no
Ocn_131	Rabaul	−4.1916	151.9772	coral	Sr/Ca	1867	1998	<1	^287^	447	no
Ocn_132	SO90-39KG/SO130-275KL	24.8333	65.9167	marine sediment	foraminifera	−117	1989	8	^288^	448	yes
Ocn_133	Coast of Portugal	41.1	−8.9	marine sediment	alkenone	971	1993	9	^289^	449	yes
Ocn_135	RAPiD-17-5P, South Iceland	61.48	−19.53	marine sediment	d18O	798	1915	14	^290^	450	yes
Ocn_136	P178-15-BC1	11.955	44.3	marine sediment	TEX86	1628	2000	9	^291^	451	yes
Ocn_137	P178-15P	11.955	44.3	marine sediment	TEX86	131	1980	15	^291^	451	yes
Ocn_138	Northeast Breakers	32.47	−64.7	coral	d18O	1519	1604	<1	^292^	452	yes
Ocn_139	Mayotte	−12.65	45.1	coral	d18O	1866	1994	<1	^246^	401	no
Ocn_140	Tongue of the Ocean, Exuma Sound Bahamas	23.766	−76.053	sclerosponge	d18O	1890	1990	1	^293^	453	no
Ocn_141	Tongue of the Ocean, Exuma Sound Bahamas	23.766	−76.053	sclerosponge	Sr/Ca	1890	1990	1	^293^	453	no
Ocn_142	Tongue of the Ocean, Exuma Sound Bahamas	23.504	−76.577	sclerosponge	d18O	1890	1990	1	^293^	453	no
Ocn_143	Tongue of the Ocean, Exuma Sound Bahamas	23.504	−76.577	sclerosponge	Sr/Ca	1890	1990	1	^293^	453	no
Ocn_144	Bermuda	30.0867	−64.5417	coral	d18O	1856	1920	3	^294^	454	no
Ocn_145	Bermuda	30.0867	−64.5417	coral	d18O	1833	1904	3	^294^	454	yes
Ocn_146	Pedra de Lume, Cape Verde Islands	16.76	−22.8883	coral	d18O	1929	2002	<1	^295^	455	no
Ocn_147	Los Roques, Venezuela	11.77	−66.75	coral	d18O	1918	2005	<1	^296^	456	no
Ocn_148	Gulf of Maine	43.6561	−69.8017	bivalve	d18O	1033	2003	1	^297^	457	yes
Ocn_149	Montego Bay Jamaica	18.4667	−77.95	sclerosponge	d18O	1344	1991	3	^298^	458	yes
Ocn_150	Montego Bay Jamaica	18.4667	−77.95	sclerosponge	Sr/Ca	1356	1991	3	^298^	458	yes
Ocn_151	Pedro Bank Jamaica	17.5333	−78.95	sclerosponge	d18O	1389	1992	5	^298^	458	no
Ocn_152	Pedro Bank Jamaica	17.5333	−78.95	sclerosponge	Sr/Ca	1384	1992	5	^298^	458	no
Ocn_153	Houtman Abrolhos	−28.4609	113.772	coral	Sr/Ca	1798	2010	1	^299^	459	yes
Ocn_154	Houtman Abrolhos	−28.4589	113.749	coral	d18O	1848	2010	1	^299^	459	yes
Ocn_155	Houtman Abrolhos	−28.4589	113.749	coral	Sr/Ca	1848	2010	1	^299^	459	yes
Ocn_156	Ifaty	−23.15	43.58	coral	d18O	1882	1994	1	^300^	411	no
Ocn_157	Ifaty	−23.3572	43.6195	coral	d18O	1905	1994	1	^300^	411	no
Ocn_158	Houtman Abrolhos Islands	−28.4667	113.7667	coral	calcification rate	1900	2010	1	^301^	1	no
Ocn_159	Clipperton	10.2773	−109.2131	coral	Sr/Ca	1935	1994	<1	^302^	460	no
Ocn_160	Clipperton	10.2773	−109.2131	coral	Sr/Ca	1894	1994	<1	^302^	460	no
Ocn_161	Clipperton	10.2773	−109.2131	coral	Sr/Ca	1942	1994	<1	^302^	460	no
Ocn_162	Clipperton	10.2773	−109.2131	coral	d18O	1874	1957	<1	^302^	460	no
Ocn_163	Clipperton	10.2773	−109.2131	coral	Sr/Ca	1874	1957	<1	^302^	460	no
Ocn_164	Coral Sea	−17.73	148.43	coral	d18O	1708	1988	5	^303^	461	yes
Ocn_165	Coral Sea	−17.73	148.43	coral	Sr/Ca	1708	1988	5	^303^	461	yes
Ocn_166	Madang Lagoon, Papua New Guinea	−5.22	145.82	coral	d18O	1923	1991	<1	^304^	462	no
Ocn_167	Xisha Island, South China Sea	16.85	112.33	coral	Sr/Ca	1906	1994	<1	^305^	463	no
Ocn_168	Buccoo Reef, Tobago	11.17	−60.85	coral	d18O	1927	1990	1	^306^	464	no
Ocn_169	Buccoo Reef, Tobago	11.17	−60.85	coral	d18O	1932	1990	1	^306^	464	no
Ocn_170	Bundegi reef, Ningaloo	−21.8333	114.1833	coral	calcification rate	1900	2010	1	^301^	1	no
Ocn_171	Clerke Reef, Rowley Shoals	−17.2667	119.3667	coral	calcification rate	1900	2010	1	^301^	1	no
Ocn_172	Coral Bay, Ningaloo	−23.0333	113.8167	coral	calcification rate	1900	2010	1	^301^	1	no
Ocn_173	Imperieuse, Rowley Shoals	−17.5167	118.9667	coral	calcification rate	1900	2010	1	^301^	1	no
Ocn_174	Tantabiddi reef, Ningaloo	−21.9	113.9667	coral	calcification rate	1900	2010	1	^301^	1	no
Ocn_175	GBR	−16.7167	146.0333	coral	d18O	1807	2004	5	^307^	465	yes
Ocn_176	GBR	−16.7167	146.0333	coral	Sr/Ca	1807	2004	5	^307^	465	yes
Ocn_177	Kiritimati	1.6792	−157.2473	coral	d18O	1938	1994	<1	^308^	466	no
Ocn_178	Rarotonga	−21.2378	−159.8278	coral	d18O	1907	2000	<1	^268^	467	no
Ocn_179	Palau	7.2708	134.3837	coral	d18O	1899	2008	<1	^309^	468	no
Ocn_180	Palau	7.2859	134.2503	coral	d18O	1793	2008	<1	^309^	468	yes
Ocn_181	Malo Channel, Espiritu Santo Island, Vanuatu	−15.7	167.2	coral	d18O	1929	1992	<1	^310^	469	no
Ocn_182	Malo Channel, Espiritu Santo Island, Vanuatu	−15.7	167.2	coral	Sr/Ca	1929	1992	<1	^310^	469	no
Ocn_183	Great Barrier Reef	−18.315	146.595	coral	Coral Sr/Ca	1568	1983	5	^311^	470	no
SAm_003	Laguna Aculeo	−33.85	−70.92	lake sediment	reflectance	856	1997	1	^312^	471	yes
SAm_006	Central Andes composite 11	−40.1	−72.05	tree	TRW	1492	1995	1	^313^	73	yes
SAm_024	Central Andes composite 6	−38.5	−71.5	tree	TRW	1435	2006	1	^314^	472	yes
SAm_025	Central Andes composite 9	−39.33	−71.25	tree	TRW	1636	2006	1	^314^	473	yes
SAm_026	Quelccaya Ice Cap	−13.9333	−70.8333	glacier ice	d18O	226	2009	1	^315^	474	yes
SAm_029	Central Andes composite 15	−41.17	−71.92	tree	TRW	1582	1991	1	^314^	475	yes
SAm_030	Laguna Chepical	−32.2667	−70.5	lake sediment	reflectance	−1161	2005	1	^316^	476	yes
SAm_031	Laguna Escondida	−45.5167	−71.8167	lake sediment	BSi	400	2008	1	^317^	477	yes
Columns are: PAGES2k identifier, Site Name, latitude (°N), longitude (°E), archive type, proxy observation, earliest year sampled, latest year sampled, resolution (defined as the median spacing between consecutive observations), publication citation, data citation, and whether the record passes the *basicFilter* criteria (Supplementary Fig. 2). A more complete list of metadata are in Supplementary Table 1.											

**Table 3:** Contents of the FigShare repository associated with this descriptor.

**Filename**	**Contents**
LoadData.md	Markdown-style text file explaining how to load the data
PAGES2k_v2.0.0_LiPD.zip	Original records in LiPD format
PAGES2k_v2.0.0.mat	Matlab-readable data structure
PAGES2k_v2.0.0-ts.pklz	Python-readable data structure
PAGES2k_v2.0.0.Rdata	R-readable data structure
HADCRUT4_median_GraphEM.mat	Mat file containing the GraphEM-infilled version of HadCRUT4.2
Africa2k_QCfig_bundle.pdf	Quality-control plots for Africa
Ant2k_QCfig_bundle.pdf	Quality-control plots for Antartica
Arc_QCfig_bundle.pdf	Quality-control plots for the Artic
Asia_QCfig_bundle.pdf	Quality-control plots for Asia
Aus_QCfig_bundle.pdf	Quality-control plots for Australasia
Eur_QCfig_bundle.pdf	Quality-control plots for Europe
NAm_QCfig_bundle.pdf	Quality-control plots for North America
SAm_QCfig_bundle.pdf	Quality-control plots for South America
Ocean_QCfig_bundle.pdf	Quality-control plots for ocean regions
Global_QCfig_bundle.pdf	Quality-control plots for all regions
