# Supplementary material for: A global multiproxy database for temperature reconstructions of the Common Era
Source: Sci Data. 2017 Jul 11;4:170088. doi: 10.1038/sdata.2017.88 (PMC5505119; doi:10.1038/sdata.2017.88)
Supplement: Supplementary Information [file sdata201788-s2.pdf]

Supplementary information to "A global multiproxy database for temperature reconstructions of the Common Era"

PAGES2k consortium

April 21, 2017

Contents

1 Tree-ring data **1**

2 Extended metadata **1**

2.1 PAGES2k metadata 1

2.2 Relationship to version 1 1

3 Alternate visualisations **2**

4 Effect of screening criteria **2**

5 Seasonal effects **8**

1 Tree-ring data

The network of tree-ring width and density records was constructed by combining publicly-available data housed by the International Tree-ring Data Bank, with data contributed by individual investigators. To be included in the PAGES2k proxy temperature database, tree-ring data were required to: (i) extend back to at least 1700 CE, and (ii) exhibit a positive and significant correlation ($p < 0.05$) to local or regional temperature (averaged over the entire year or over the growing season). The final set of 416 tree-ring records is mainly drawn from high-elevation or high-latitude sites, and is clustered principally in the western mountains of North America, the northern edge of the pan-Arctic boreal forest, and the mountains of central Asia. This global multiproxy dataset includes fewer tree-ring records than some previous multiproxy synthesis efforts [e.g. 1] that included records without regard to their temperature sensitivity. Because many trees are more strongly influenced by moisture availability than by growing season temperatures [2], including only those records sensitive to temperature substantially reduces the overall number of tree-ring records. Furthermore, restricting this compilation to trees that are positive responders (where warmer temperatures lead to enhanced tree growth) excludes records that exhibit a significant and negative association with temperature as a secondary effect of moisture stress.

Tree-ring series from North America, including those used by the PAGES2k Consortium [3], have now all been standardized with a negative exponential function [4–7]. Tree-ring series from other regions have kept the standardization used in the original publication. Unlike [3], in the present version, tree-ring records include only ring-width or density measurements rather than the reconstructions derived from them. Therefore, many of the North American dendroclimatological records used in [3] are no longer employed. Also, in the North American component of [3], unlike the current version, tree-ring data were screened and incorporated into the North American temperature reconstructions as the leading principal components of the tree-ring chronologies utilized. The rationale, methodological detail, and associated reconstruction performance metrics for that usage are described in the supplemental information in [3] (cf. section 4a).

Although the network of tree-ring chronologies currently included in the PAGES2k proxy temperature database are limited to ring width and density, future versions of the database will likely include tree-ring chronologies based on different properties such as isotopes, cell wall thickness, tracheid diameters, microfibril angle, blue intensity, vessel area and cell numbers, amongst others.

2 Extended metadata

Because of page-format restrictions, Table 1 includes only a subset of metadata for each record. This Supplement includes two additional tables.

2.1 PAGES2k metadata

Supplemental Table S1 includes the essential metadata fields from the current database. The table is available digitally (`PAGES2k_2.0.0_TableSupp.xlsx`, tab Table S1).

2.2 Relationship to version 1

Supplemental Table S2 lists all of the records from previous versions of the PAGES2k proxy temperature dataset, whether they are included in this version (2.0.0), or why they were rejected. The table is available digitally (`PAGES2k_2.0.0_TableSupp.xlsx`, tab Table S2).

Table S1: **PAGES2k extended metadata**. Properties for each record include the PAGES2k identifier assigned for this data product, the identifier used in previous PAGES2k products by the Ocean2k working group McGregor *et al.* [8] and Tierney *et al.* [9], or by PAGES2k Consortium [3], whether the record is superseded by another in this version, the archive type, the primary publication citation, its associated digital object identifier (DOI; if one exists), the secondary publication citation and DOI, the URL where the data were archived by the original author, the associated data citation, the geographic coordinates (latitude, longitude, elevation), the name of the site, the ISO 3166-1 standard name of the country/ocean basin where it is located, the earliest and latest years covered by the record, resolution of the time series (median spacing between consecutive observations), the type of proxy observation, the name of the variable used as the temperature-sensitive time series and its units, the physical feature whose temperature is sensed by the proxy (e.g. surface air temperature, sea-surface temperature), the part of the seasonal cycle recorded by the proxy (if numeric, indicates calendar month), the direction of the relationship between the proxy and temperature (positive or negative), quality control (QC) comments, initials of PAGES2k Consortium author who performed QC certification (see main text for the full names), and a permalink to the dataset's page at the NCEI-Paleo/World Data Service for Paleoclimatology.

Table S2: Crossover between previous PAGES2k data products [3, 8, 9] and version 2 of the PAGES2k temperature database [1]. Columns are: PAGES2k region, record ID in previous versions, record ID in version 2, superseded by, criterion for excluding from this version.

3 Alternate visualisations

Site locations are visualized in Figs. 1, 2 (main text) as well as quality-control figures (`Global_QCfig_bundle.pdf`, Data Citation 1). Given the dataset's global nature, no projection can do justice to proxies in all regions. Fig. S1 provides three other projections focused on the Arctic, Antarctic, and tropical oceans.

4 Effect of screening criteria

This section depicts the evolution of the PAGES2k proxy temperature network when successively more stringent levels of screening are applied. The baseline for this comparison is Fig. 1 (main text), which gathers all 692 records.

Fig. S2 displays the records that remain after applying these basic quality-control criteria (*basicFilter*): no borehole records, at least 20 points over the Common Era, going back to at least 1850 CE. These records are the ones entering some of the timeseries summaries of Fig. 7 (main text).

Next, the network is further whittled down by applying regional screening of the 600 calibratable proxies (Fig. S3), regional screening controlling for the False Discovery Rate (Fig. S4), and local screening (Fig. S5) criteria. Each set is a subset of the previous.

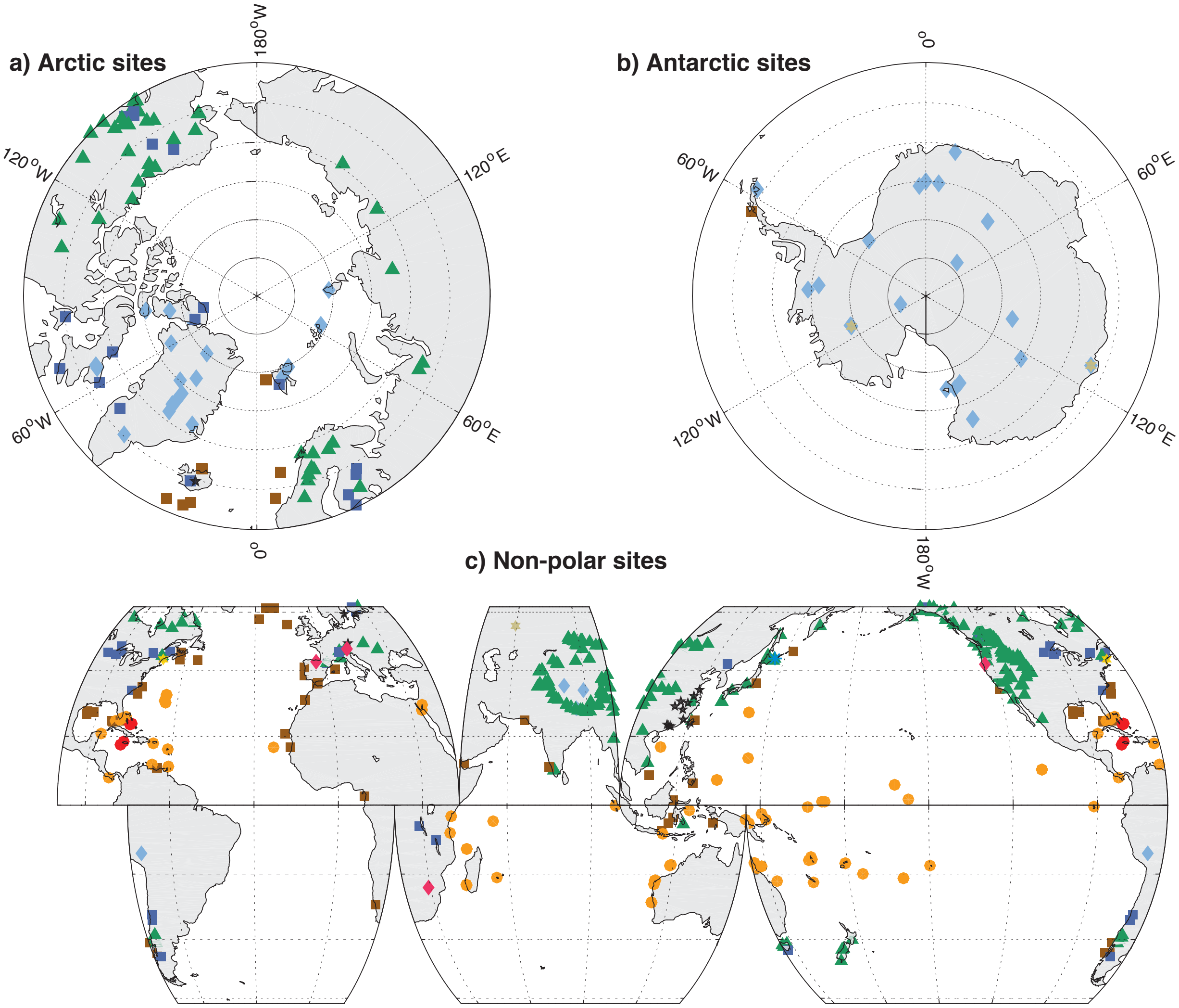

Figure S1: Alternate visualisations of PAGES2k sites and archive type. a) Arctic-centric stereographic projection; b) Antarctic-centric stereographic projection; c) Interrupted Mollweide projection, focused on tropical regions.

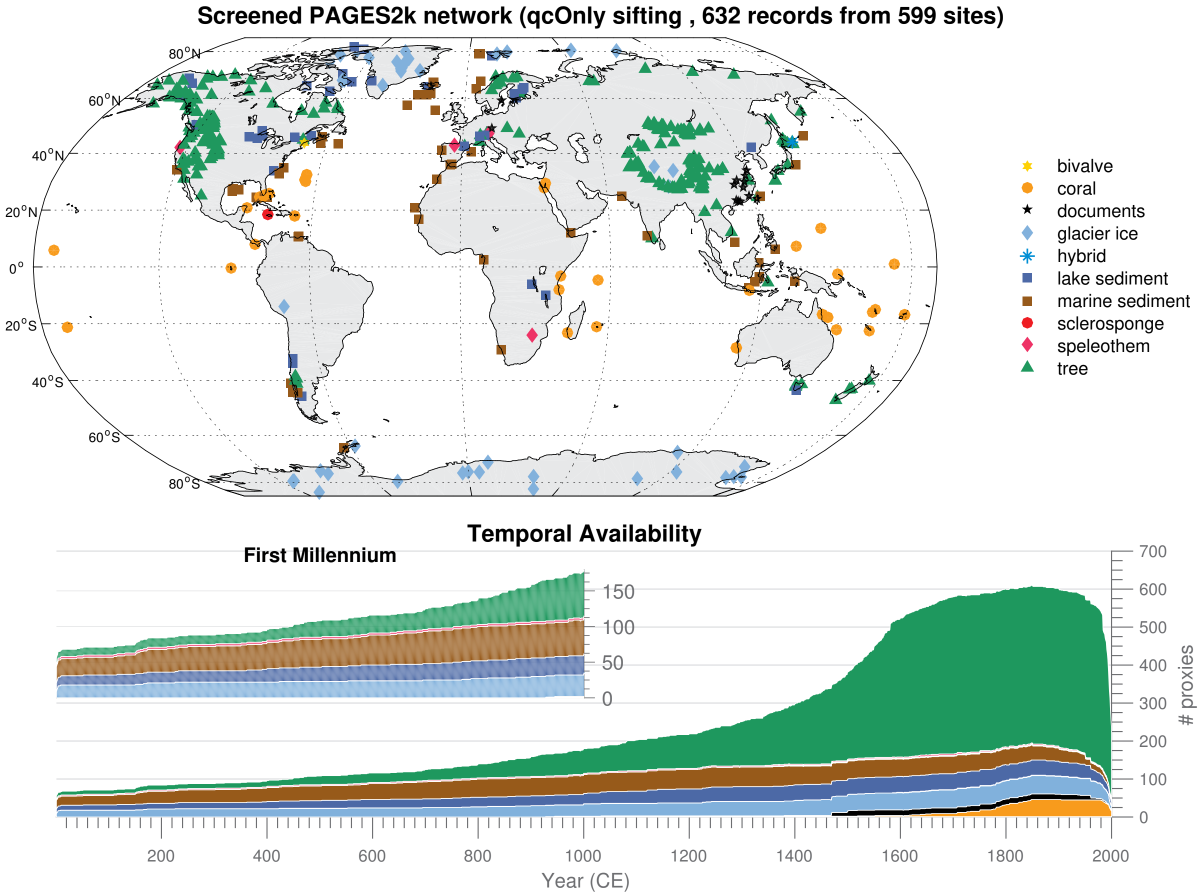

Figure S2: Records included according to the *basicFilter* used in the main text

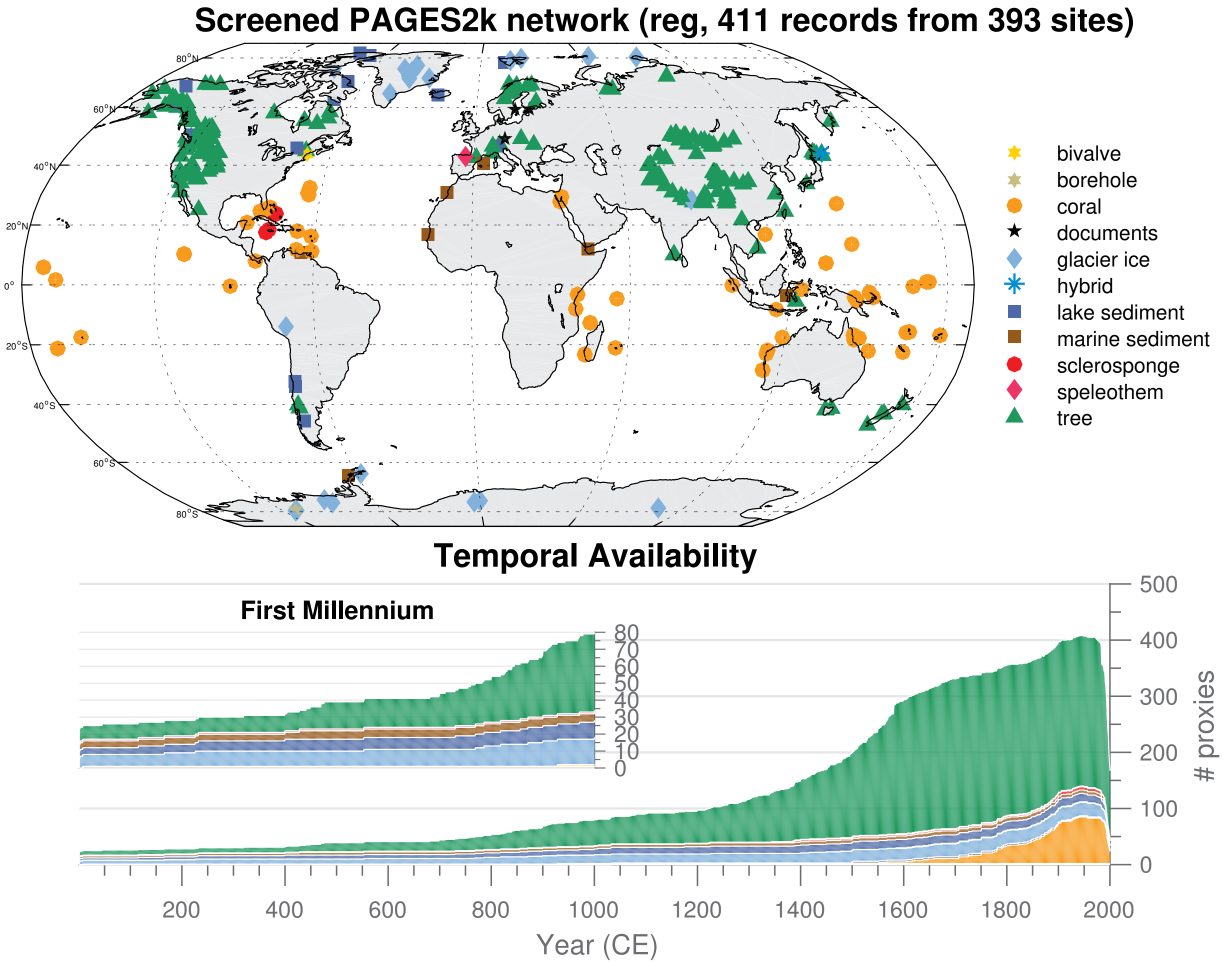

Figure S3: Database composition after screening for correlations in MAT within a 2000 km radius (compare to main text, Fig. 1a,c)

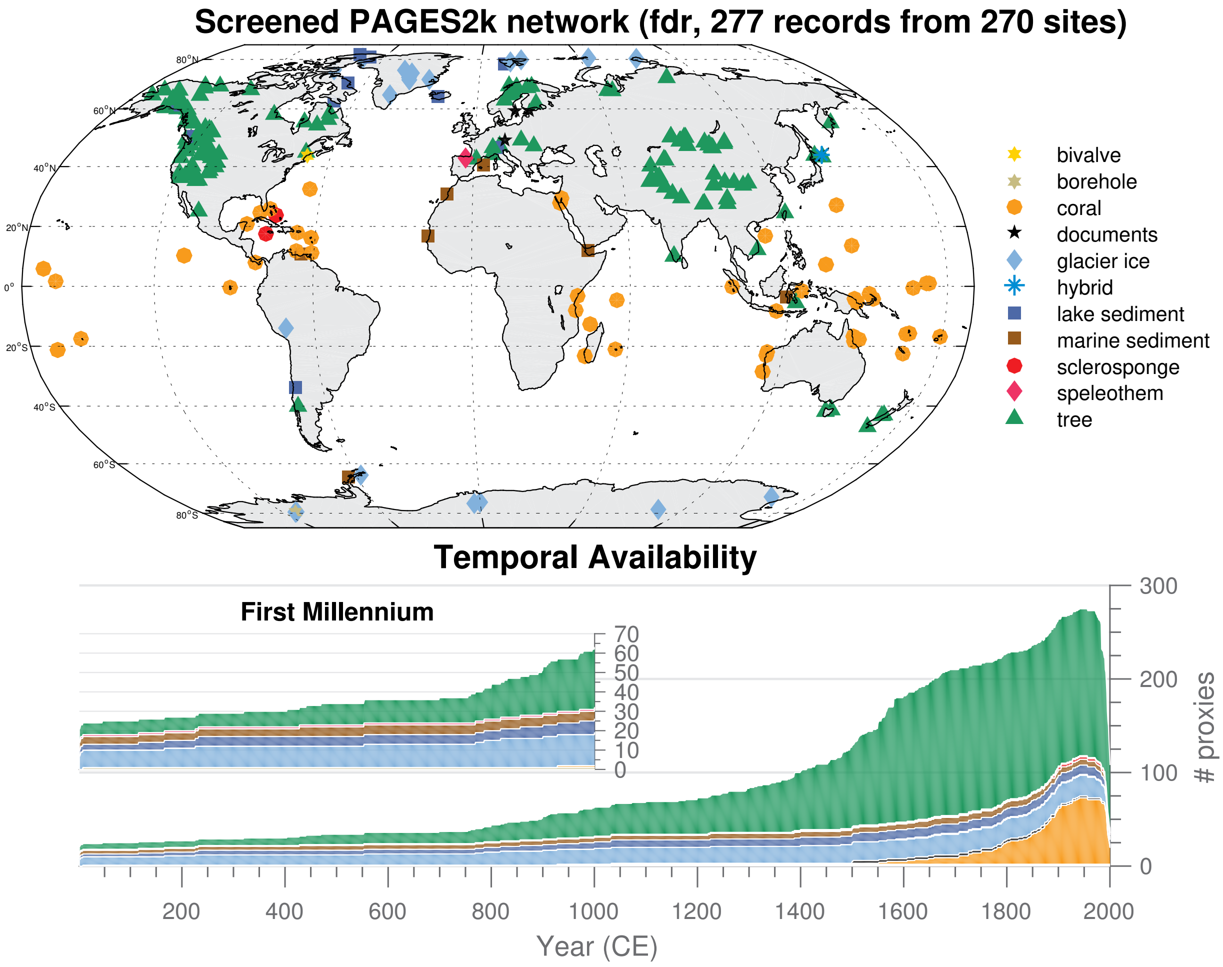

Figure S4: Same as Fig. S3, controlling for the false discovery rate.

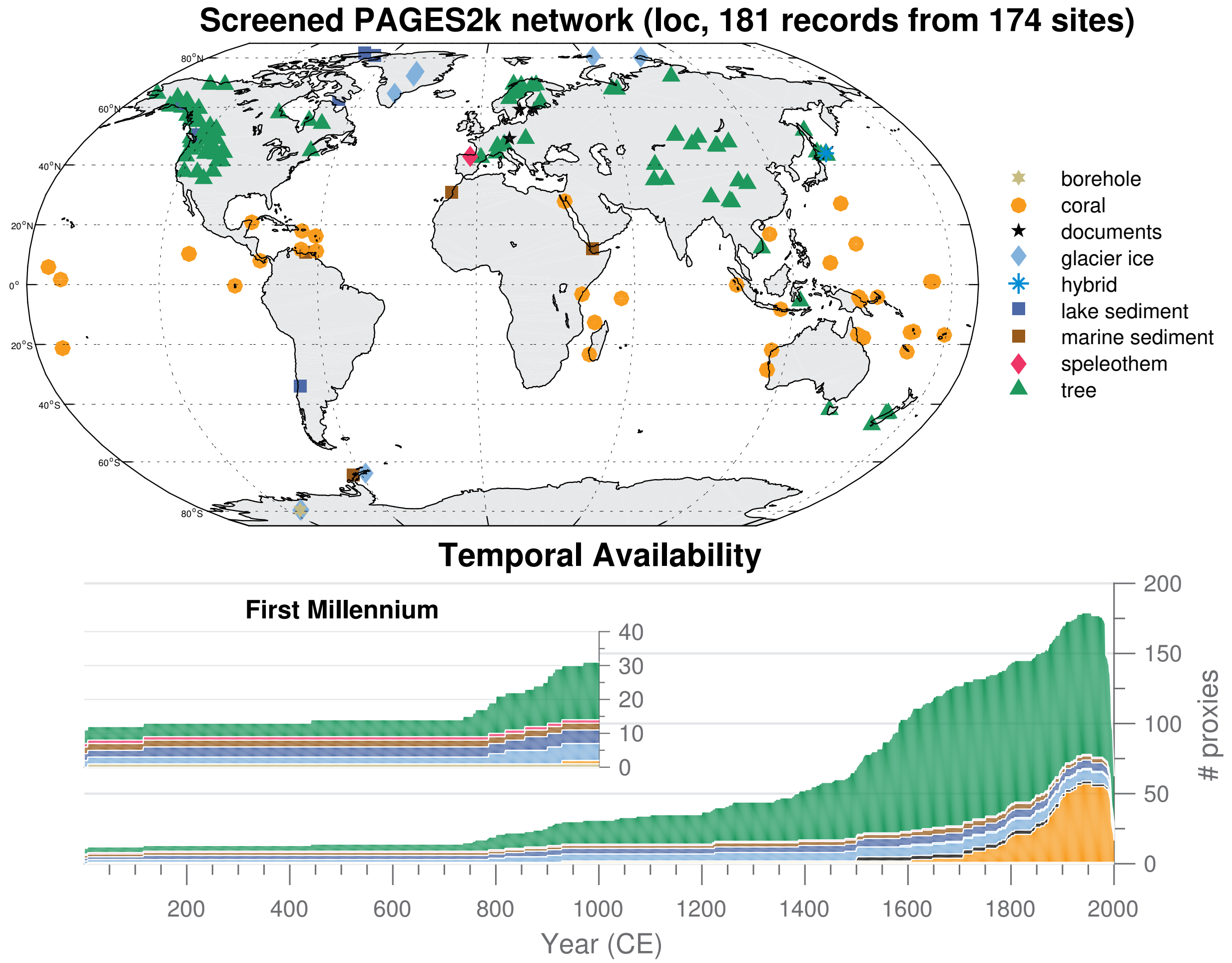

Figure S5: Same as Fig. S3, but screening for local correlations in MAT (compare also to Fig. S4 and main text, Fig. 1)

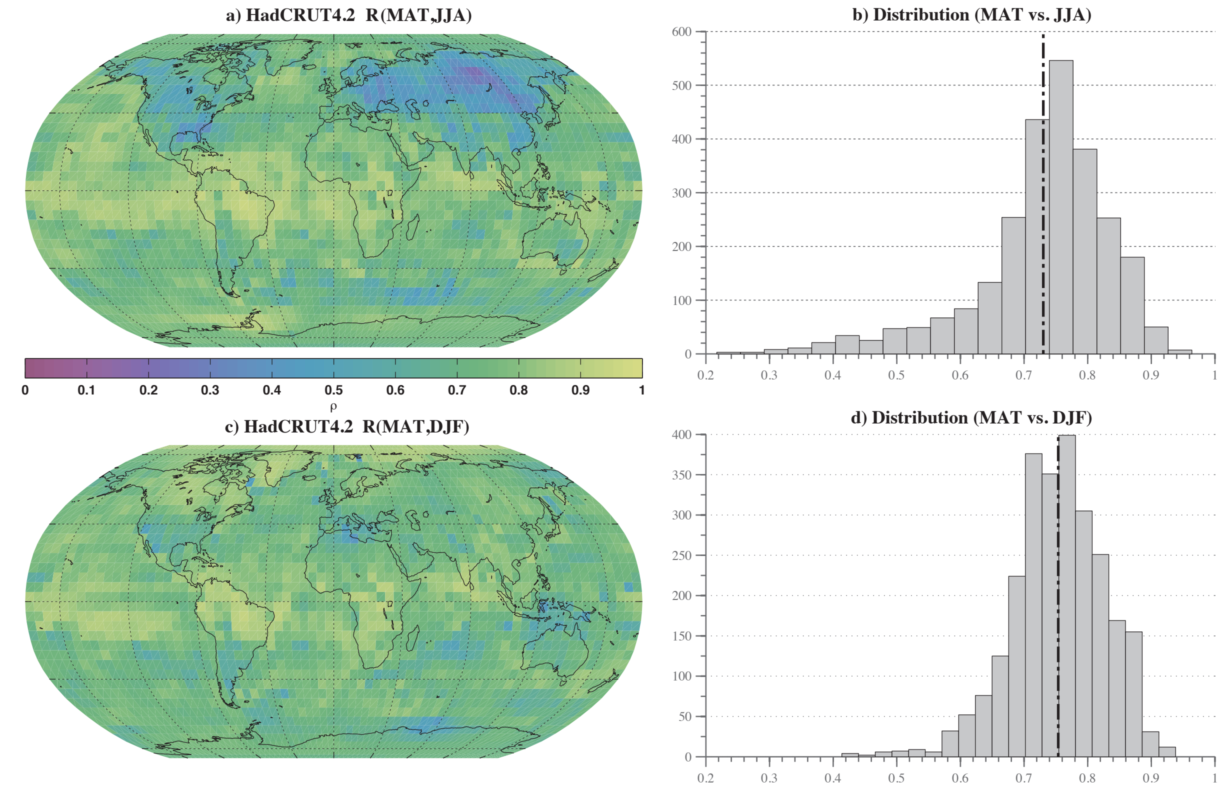

Figure S6: Contributions of extreme seasons (boreal winter and summer) to the annual average. (a) Pearson product-moment correlation coefficient of mean annual temperature with June-July-August temperature in the infilled HadCRUT4.2 dataset; (b) distributions of these correlations. (c,d): same as (a,b) but for December-January-February. In (b) and (d), dash-dotted lines indicate the mean of each distribution.

5 Seasonal effects

The extent to which proxies are informative of temperature is known to depend, sometimes very strongly, on the portion of the annual cycle which they preferentially sample [10]. Thus, before using seasonally-dependent proxies to reconstruct mean-annual temperature, one must ascertain the relationship between seasonal averages and the annual mean.

Fig. S6 explores how much of the mean annual temperature (MAT) signal can be explained by boreal summer (JJA, top) vs winter (DJF, bottom) averages. JJA-MAT correlations are very high in the tropics, where the MAT range is small, and low in the extra-tropics, particularly over Northern Hemisphere continental interiors, where the MAT range is large and dominated by winter synoptic variability. This means that proxies that preferentially record summer conditions may be adequate predictors of the annual mean in the tropics but (all other things being equal) less so over Northern Hemisphere continents. Extratropical winter variability is known to dominate the annual average, so correlations tend to be highest in the winter hemisphere for each season.

References

1. Mann, M. E. *et al.* Global signatures and dynamical origins of the Little Ice Age and Medieval Climate Anomaly. *Science* **326,** 1256–1260 (2009).
2. St George, S. An overview of tree-ring width records across the Northern Hemisphere. *Quaternary Science Reviews* **95,** 132–150 (2014).
3. PAGES2k Consortium. Continental-scale temperature variability during the past two millennia. *Nature Geoscience* **6,** 339–346 (2013).
4. Fritts, H. C. *Tree Rings and Climate* (Academic Press, New York, 1976).
5. Cook, E. R. *A Time Series Approach to Tree-Ring Standardization* PhD thesis (University of Arizona, Tucson, AZ, USA, 1985).
6. Cook, E., Briffa, K., Shiyatov, S. & Mazepa, V. *Tree-ring standardization and growth-trend estimation* in *Methods of dendrochronology: applications in the environmental sciences* (eds Cook, E. & Kairiukštis, L.) (Kluwer Academic Publishers, Netherlands, 1990), 104–123.
7. Cook, E. R., Briffa, K. R., Meko, D. M., Graybill, D. A. & Funkhouser, G. The segment length curse in long tree-ring chronology development for paleoclimatic studies. *Holocene* **5,** 229–237 (1995).
8. McGregor, H. V. *et al.* Robust global ocean cooling trend for the pre-industrial Common Era. *Nature Geoscience* **8,** 671–677 (2015).
9. Tierney, J. E. *et al.* Tropical sea surface temperatures for the past four centuries reconstructed from coral archives. *Paleoceanography* **30,** 226–252 (2015).
10. Jones, P. *et al.* High-resolution palaeoclimatology of the last millennium: a review of current status and future prospects. *The Holocene* **19,** 3–49 (2009).

Data citations

1. PAGES 2k Consortium. *A global multiproxy database for temperature reconstructions of the Common Era* `https://doi.org/10.6084/m9.figshare.c.3285353` (2017).
